# Crystal landscape in the orcinol:4,4′-bipyridine system: synthon modularity, polymorphism and transferability of multipole charge density parameters

**DOI:** 10.1107/S2052252513024421

**Published:** 2013-10-01

**Authors:** Ritesh Dubey, Mysore S. Pavan, Tayur N. Guru Row, Gautam R Desiraju

**Affiliations:** a Solid State and Structural Chemistry Unit, Indian Institute of Science, C. V. Raman Avenue, Bangalore 560 012, India

**Keywords:** cocrystal, supramolecular synthon, crystallization, crystal structure prediction, hydrogen bond

## Abstract

Polymorphism in the orcinol:4,4′-bipyridine cocrystal system is analyzed in terms of a robust convergent modular phenol⋯pyridine supramolecular synthon. Employing the *Synthon Based Fragments Approach* (SBFA) to transfer the multipole charge density parameters, it is demonstrated that the crystal landscape can be quantified in terms of intermolecular interaction energies in the five crystal forms so far isolated in this complex system. There are five crystal forms. The first has an open, divergent O—H⋯N based structure with alternating orcinol and bipyridine molecules. The other four polymorphs have different three-dimensional packing but all of them are similar at an interaction level, and are based on a modular O—H⋯N mediated supramolecular synthon that consists of two orcinol and two bipyridine molecules in a closed, convergent structure. The SBFA method, which depends on the modularity of synthons, provides good agreement between experiment and theory because it takes into account the supramolecular contribution to charge density. The existence of five crystal forms in this system shows that polymorphism in cocrystals need not be considered to be an unusual phenomenon. Studies of the crystal landscape could lead to an understanding of the kinetic pathways that control the crystallization processes, in other words the valleys in the landscape. These pathways are traditionally not considered in exercises pertaining to computational crystal structure prediction, which rather monitors the thermodynamics of the various stable forms in the system, in other words the peaks in the landscape.

## Abstract 2

The role of the supramolecular synthon as the operational structural unit in the late stages of the crystallization event is highlighted with reference to polymorphs and pseudopolymorphs in the orcinol–bipyridine cocrystal system.

## 1. Introduction   

Crystal engineering is concerned with the development of logical design strategies based on the concept of the supramolecular synthon (Desiraju, 1995 ▶) and the execution of such strategies to obtain entire families of related crystal forms of a series of chemically similar molecules. The purpose of obtaining these engineered structures is to achieve physical and chemical properties of interest and utility (Desiraju, 1989 ▶; Desiraju *et al.*, 2011 ▶). At a more fundamental level, crystal engineering may be reduced to elucidating the mechanism of crystallization (Weissbuch *et al.*, 2003 ▶; Erdemir *et al.*, 2009 ▶). Given any molecular structure, what is the crystal structure that would be obtained? If this question could be answered fully, the essential problem of crystal engineering would be solved because any pre-desired crystal structure could then be obtained at will. However, it is not likely that such an answer will be available anytime soon. The issues involved in the aggregation of molecules into clusters, larger ensembles and finally the events that lead up to nucleation and beyond are still way too complex to be addressed experimentally or computationally, in any general sense. Crystallography provides images of the ‘final’ outcomes of the crystallization event, but the constraints of long-range periodicity that are implicit for any species that gives a three-dimensional diffraction pattern hardly reveal the multiplicity and variety of chemical events that have taken place before the crystal is obtained. Perhaps there is still some justification in Ruzicka’s dismissal of solids as chemical cemeteries (Dunitz *et al.*, 1988 ▶).

Still, and even within the limits imposed by diffraction-based crystallography, one might explore a small portion of the structural panorama that just precedes the ‘final’ crystal because there are several higher energy crystal forms that may be isolated and characterized with crystallography that provide a hint about the mechanism of crystallization, at least in the later stages (Davey *et al.*, 2006 ▶; Kulkarni *et al.*, 2012 ▶; Hunter *et al.*, 2012 ▶; Davey *et al.*, 2013 ▶). These forms could include polymorphs with higher values of *Z*′, various solvates, kinetically labile species and other metastable and higher energy forms of the compound in question (Mukherjee *et al.*, 2011 ▶; Braun *et al.*, 2012 ▶). Taken collectively, one might envisage these forms as constituting a kind of landscape that profiles the structural and energetic changes that take place during the late stages of crystallization of an organic compound (Gavezzotti, 2003 ▶; Blagden & Davey, 2003 ▶; Price, 2008 ▶). Some of us have shown recently, using the example of fluoro-substitution in benzoic acids, that subtle chemical variation of a molecular scaffold permits the exploration of structural space that would otherwise be experimentally inaccessible (Dubey *et al.*, 2012 ▶).

The formation of two-component molecular crystals, or cocrystals (Desiraju, 2003 ▶; Dunitz, 2003 ▶), is a well researched aspect of modern crystal engineering (Herbstein, 2005 ▶; Bond, 2007 ▶; Stahly, 2009 ▶; Wouters *et al.*, 2011 ▶), although the phenomenon itself has been known since the isolation of quinhydrone more than 150 years ago (Wöhler, 1844 ▶). An interesting aspect of recent research on cocrystals, and indeed this was hinted at more than a decade ago when cocrystals came into the foreground, is that they may be less prone to form polymorphs than single-component crystals (Vishweshwar *et al.*, 2005 ▶). This type of thinking possibly arose from the idea that cocrystal formation is only possible if very specific interactions between the two components are optimized and as such, these substances are less likely to form multiple crystal forms. Of course, such a contention can hardly be proved or disproved because it is, in Zaworotko’s words, like ‘proving the negative’ (Almarsson & Zaworotko, 2004 ▶). However, there has always been an interest in this matter. Recently, one of us co-authored a report on two polymorphs of the 2:3 cocrystal of orcinol (5-methylresorcinol) and 4,4′-bipyridine, **I** and **II** (Tothadi *et al.*, 2011 ▶). Subsequently, the present group of authors were able to isolate two more polymorphs, **III** and **IV**, and one 1:1 cocrystal, **V**, which might be termed pseudopolymorphs[Chem scheme1]. Noting that it was quite unusual to obtain five crystal forms in a cocrystal system, a systematic investigation of these forms was initiated, in the context of the structural landscape. In the course of this study, it was noted that the five forms are related through some basic supramolecular synthons and this confers a certain element of modularity (Desiraju, 2010 ▶; MacGillivray *et al.*, 2000 ▶) in these crystal structures. We have previously shown that the modularity of the supramolecular synthon is responsible for the successful transferability of charge density derived multipole parameters for structural fragments, thus creating a possibility for the derivation of charge density maps for new compounds, in effect opening up an opportunity for the large scale application of charge density maps as a general structural tool in crystal engineering. We termed this methodology the *Supramolecular Synthon Based Fragments Approach* (SBFA) (Hathwar, Thakur, Row *et al.*, 2011 ▶). We also showed that the SBFA method is applicable not only to single-component crystal structures but also to two-component crystals, or cocrystals (Hathwar, Thakur, Dubey *et al.*, 2011 ▶). The SBFA approach is applied here to the crystal forms in the present study, in other words to polymorphs of cocrystals. The purpose of the transferability was to quantify the various intermolecular interactions present in the different polymorphic forms of the crystal landscape of the multi-component system. In effect, the utility of transferability of multipole parameters among the robust synthons in the various polymorphic modifications in cocrystals is demonstrated. The link between charge density distribution associated with transferable synthons and the possible aggregation pathways indicated in the landscape offers a unique possibility to quantify intermolecular interaction energies associated with kinetically stable polymorphic forms.
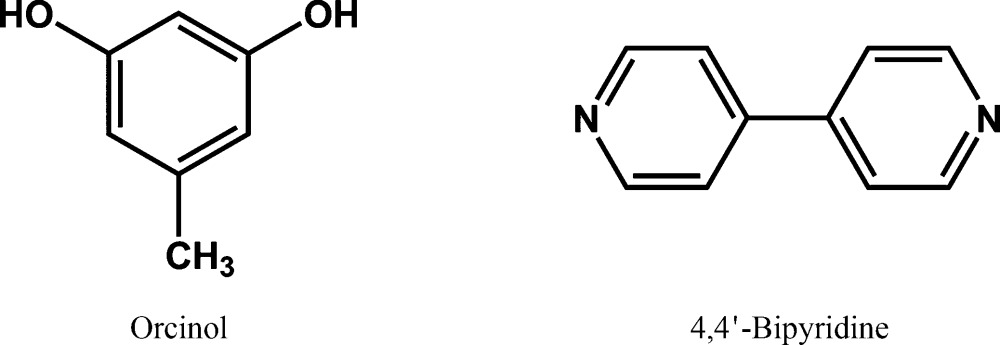


## 2. Experimental   

### 2.1. Materials   

Orcinol was purchased from Sigma Aldrich and 4,4′-bipyridine from Alfa Aesar and used without further purification. For the crystallization of all compounds, several stoichiometric ratios such as 1:1, 1:2, 2:1 and 2:3 were tried along with various crystallization methods such as solvent evaporation, sublimation and use of anti-solvent. After a week, good quality single crystals, which were suitable for the single-crystal diffraction experiments, were obtained. The ratio of the two compounds obtained in the crystal is not necessarily the ratio in which they are taken for the crystallization. Table 1 ▶ gives salient details of the cocrystals investigated in this study. Despite several attempts, it was not possible to obtain cocrystal **I** again. It may be noted that form **III** is obtained *via* sublimation, a technique that is not generally customary for multi-component crystals.

### 2.2. Data collection and structure refinement details   

Routine data sets for compounds **II**, **IV** and **V** were collected at 100 K on an Oxford Xcalibur diffractometer with a microfocus X-ray source (Mo *K*α), equipped with a Cryojet-HT nitrogen gas-stream cooling device. The variable-temperature data sets for **III** were collected at 293, 200, 160, 140 and 120 K. In all these cases, data were processed with *CrysAlisPro* (Oxford Diffraction, 2011 ▶). Structure solution and refinements were performed with *SHELX2012* (Sheldrick, 2008 ▶) using the *WinGX* suite (Farrugia, 2012 ▶).

### 2.3. High-resolution charge density data collection and structure refinement details of 4-hydroxybenzoic acid:isonicotinamide cocrystal   

These data provide the required O—H⋯N synthon data entry into the in-house library which can be used for the subsequent analysis of the polymorphs of orcinol:bipyridine. Data were collected on a single crystal of reasonable size and quality (as was examined under a polarizing microscope) which was affixed to a Hampton Research cryoloop using Paratone-N oil. The crystal was cooled to 100 K with a liquid nitrogen stream using an Oxford cryosystems N_2_ open-flow cryostat. High-resolution X-ray data up to (sinθ/λ)_max_ = 1.08 Å^−1^ with redundancy (∼ 14) and completeness (∼ 100%) were collected on a Bruker Kappa Apex II CCD diffractometer using Mo *K*α radiation at 100 K. Data collection strategies were generated using the *COSMO* module of the Bruker software suite (Bruker, 2006 ▶). The crystal-to-detector distance was fixed at 40 mm and the scan width was 0.5° per frame during the data collection. Cell refinement, data integration and reduction were carried out using the *SAINTPLUS* program. Numerical absorption correction was done by crystal face indexing. Sorting, scaling and merging of the collected data sets were carried out using the *SORTAV* program (Blessing, 1997 ▶). The crystal structure was solved by direct meth­ods and refined in the spherical-atom approximation using *SHELXL2012* (Sheldrick, 2008 ▶) from the *WinGX* suite (Farrugia, 2012 ▶). The crystallographic information and multipole refinement details are provided in the supporting information.

### 2.4. Transferability of multipole parameters using the SBFA   

**Figure 1:**
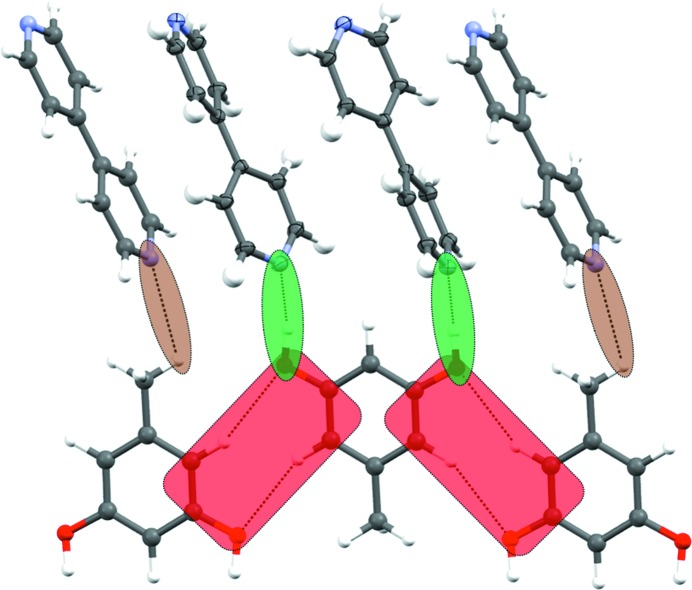
Logical fragments based on supramolecular synthons (color shaded) in forms **II** through to **V**. Notice the brown synthon which consists only of a weak C—H⋯N interaction. These fragments may be transferred from structure to structure to generate a ‘synthetic’ charge density map.

Polymorphs of orcinol (5-methylresorcinol) and 4,4′-bipyridine studied in the present work were divided into chemically reasonable molecular fragments based on their supramolecular environments (supramolecular synthons; Fig. 1 ▶). The refined multipole parameters (*P*_val_, *P*_lm_, κ and κ′) present in the in-house library of experimental charge density data sets were used for SBFA transferability to all these target molecules. Scaling and initial refinement of the positional and displacement parameters of all atoms were carried out using the *XD2006* package (Volkov *et al.*, 2006 ▶). The H atoms were fixed to neutron values and the anisotropic displacement parameters of H atoms were computed using the *SHADE2* server (Madsen, 2006 ▶; Munshi *et al.*, 2008 ▶). Charge neutralization was obtained by fixing the individual atomic monopole to neutral atom values, followed by the refinement of atomic monopoles for all atoms which allowed realistic atomic charge values to be obtained. All other multipole parameters including κ and κ′ were kept fixed during the refinements.

### 2.5. Theoretical evaluation of charge density to authenticate the multipole parameters derived from SBFA   

Single-point periodic quantum mechanical calculations at the B3LYP/6-31G(d,p) level were carried out using *CRYSTAL09* (Dovesi *et al.*, 2009 ▶) with the neutron-normalized geometries obtained from experimental structure refinement. The shrinking factors (IS1, IS2 and IS3) along with the reciprocal lattice vectors were set to 4 (30 *k*-points in the irreducible Brillouin zone). The bi-electronic Coulomb and exchange series values for the truncation parameter were set as ITOL1 − ITOL4 = 8 and ITOL5 = 17, respectively, for the *CRYSTAL09* calculations. The level shifter was set to 0.7 Hartree per cycle. The self-consistent field convergence limit was chosen as ∼ 10^−7^ Hartree. The cohesive energy calculation was performed in all cases and the Grimme dispersion corrections along with the basis set superposition error corrections were included in the calculations. For a definition of the cohesive energy and details of its calculation refer to the supporting information. Theoretical structure factors obtained from the *CRYSTAL09* single-point calculations for **II** and **III** were used in the multipole refinements using the XD software package (Volkov *et al.*, 2006 ▶). Molecular geometry and the atomic displacement parameters for all atoms were kept fixed throughout the multipole refinement of the static model. Refinements and analysis of the theoretically obtained charge density data were performed with an unrestricted multipole model to compare the results from the transferred SBFA model. The purpose of the theoretical modeling in the above two cases was to benchmark the quality of SBFA modeled densities.

## 3. Results and discussion   

### 3.1. Analysis of crystal forms   

**Figure 2:**
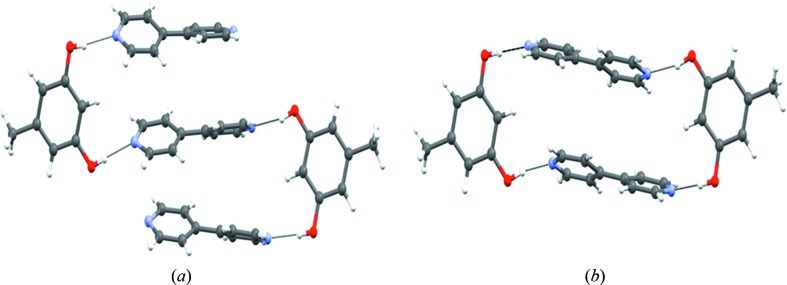
Divergent and convergent arrangement of O—H⋯N hydrogen bonds in (*a*) form **I** (synthon **A**), and (*b*) forms **II**–**V** (synthon **B**) of orcinol:4,4′-bipyridine cocrystals.

All five solid forms of orcinol–bipyridine are characterized by O—H⋯N hydrogen bonds between the two components (Fig. 2 ▶). These hydrogen bonds form a finite divergent pattern (synthon **A**) that consists of two orcinol molecules and three bipyridine molecules, seen in form **I**, or a closed convergent pattern (synthon **B**) that consists of two orcinol and two bipyridine molecules, seen in the related forms **II** through to **V**. The latter pattern was first identified by MacGillivray in his extensive studies of solid-state topochemical reactions of phenol–pyridine cocrystals (Gao *et al.*, 2004 ▶; MacGillivray *et al.*, 2008 ▶; MacGillivray, 2008 ▶). Because synthon **B** is a zero-dimensional entity, it is possible that it persists in solution. Still, both divergent and convergent possibilities seem to be efficient molecular arrangements that use four O—H⋯N hydrogen bonds each. These assemblies are further supported by weak intermolecular interactions such as C—H⋯N, C—H⋯O, C—H⋯π and π⋯π interactions.

**Figure 3:**
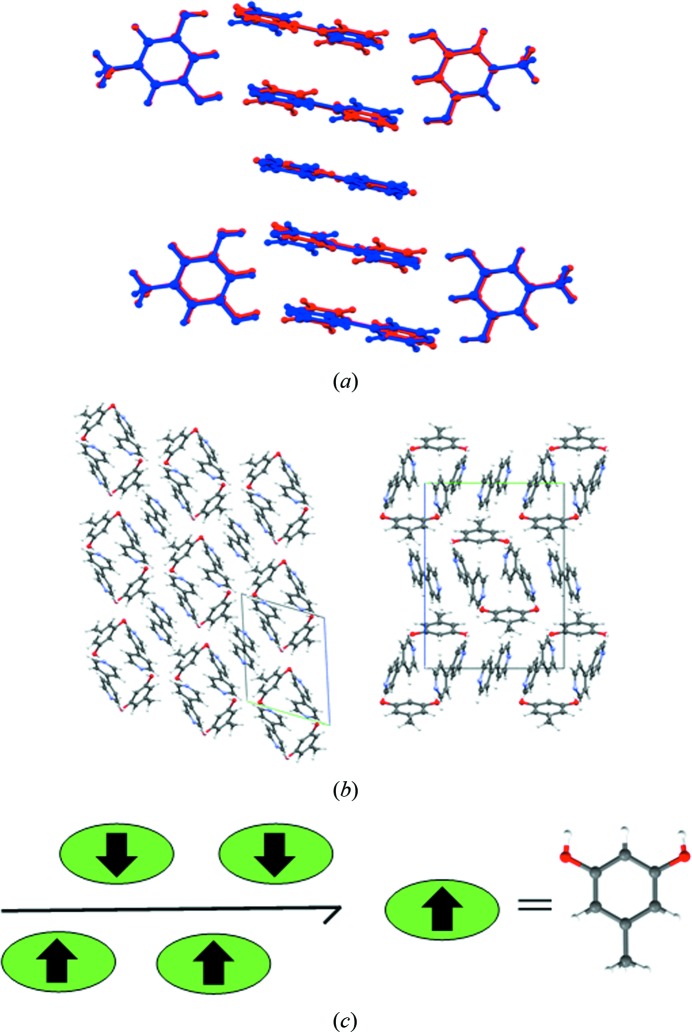
Orcinol–bipyridine: (*a*) overlay diagram of forms **II** and **III**; (*b*) arrangement of LSAMs in form **II**, right, and form **III**, left; (*c*) schematic of molecular arrangement along the2_1_-screw axis in form **III**.

In the triclinic form **II** (Fig. 3 ▶ *b*), the O—H⋯N hydrogen bonds in synthon **B** are normal (*d*, θ; 1.74 Å, 177.5°; 1.77 Å, 168.8°) and the arrangement is supported by weak C—H⋯N interactions (2.63 Å, 123.1°) from the orcinol C—H. The structure also contains a ‘free’ bipyridine molecule which is sandwiched between two hydrogen-bonded tetramers (that is, synthon **B**) and stabilized by π⋯π and C—H⋯N interactions (2.62 Å, 148.5°) from bipyridine C—H.

The structure of form **II** is closely related to that of the new monoclinic form **III** (*P*2_1_/*n*), which is also a 2:3 cocrystal. The tetramer synthons **B** (O—H⋯N, 1.75 Å, 175.2°; 1.76 Å, 175.3°) sandwich the free bipyridine in nearly the same manner. This larger assembly consisting of two tetramers and the sandwiched bipyridine is termed a *Long Range Synthon Aufbau Module* (LSAM) (Ganguly & Desiraju, 2008 ▶, 2010 ▶). The LSAM is a late synthon and Fig. 3 ▶(*a*) shows that the LSAMs in forms **II** and **III** are exceedingly similar. The advantage in differentiating between small and large synthons lies in the fact that the small synthons do not serve to distinguish well between polymorphs – the larger synthons include degrees of structural detail that permit such an exercise. In other words, the dissimilarity between the forms pertain not to the hydrogen bonding itself but rather to the arrangement of the LSAMs with respect to the crystallographic axes (Fig. 3 ▶ *b*). All this clearly indicates that at a molecular recognition level (small supramolecular synthons) both forms are nearly the same, but as the molecular assembly becomes increasingly larger, the forms become different (Fig. 3 ▶ *b*). Incidentally, orcinol molecules are nearly parallel to the crystallographic *b*-axis in form **III**. Taken with the mutually perpendicular arrangement of orcinol and bipyridine molecules, this leads to a pseudo 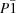 character for the *P*2_1_/*n* structure (Fig. 3 ▶ *c*) (Sarma & Desiraju, 1986 ▶).

**Figure 4:**
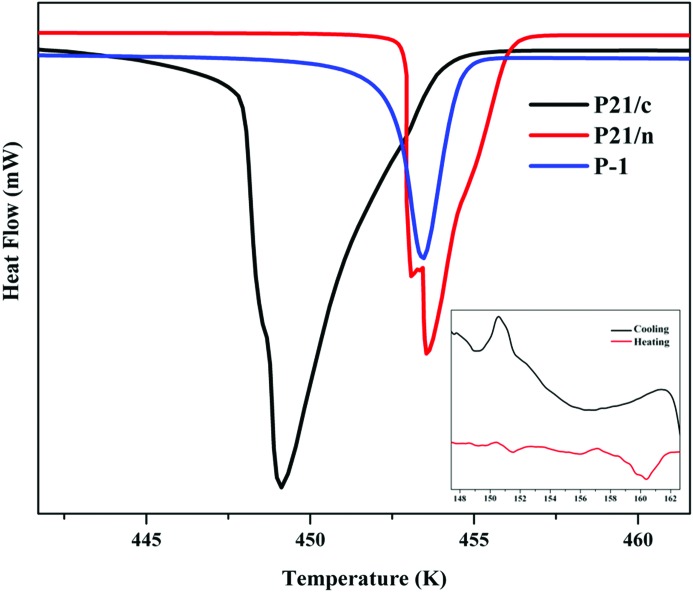
Thermodynamic (DSC) profile of forms **II**, **III** and **V**. Low temperature reversible **III** → **IV** phase transition (inset).

**Figure 5:**
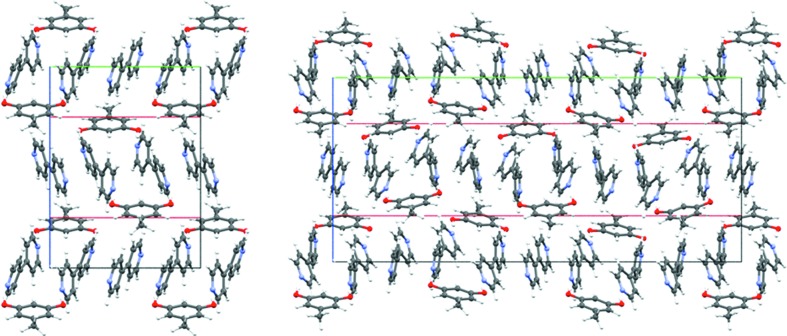
Orcinol–bipyridine: structure along the unique axis (shown in red) in forms **III** (left) and **IV** (right). Notice the triple modulation in the low temperature form.

We collected single-crystal data of form **III** at five different temperatures and this showed evidence of a reversible phase transition between 140 and 160 K (Fig. 4 ▶). The structural details are given in the supporting information. After cooling through the phase transition, form **III** provides a new low-temperature crystal structure, form **IV**, which is modulated along the unique axis so that it is nearly three times the value of that in form **III** (Fig. 5 ▶). The O—H⋯N hydrogen bonds (1.74 Å, 173.9°; 1.74 Å, 176.8°; 1.75 Å, 170.6°; 1.76 Å, 171.9°; 1.77 Å, 177.3°) are again normal and there are also some other weak interactions. Form **IV** has a better packing than form **III** as shown from the Kitaigorodskii Packing Indices (KPI) (Table 2 ▶).

**Figure 6:**
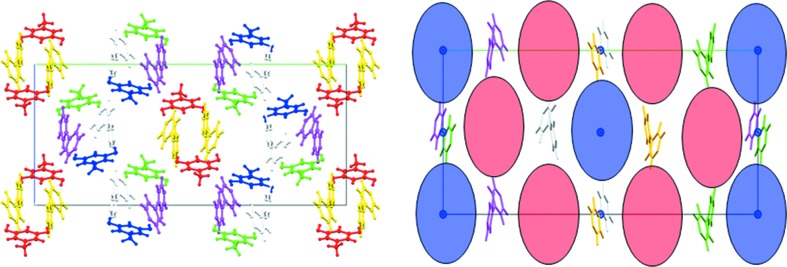
Arrangement of center and pseudo-center related synthons in form **IV**. Color coding is based on symmetry equivalence: blue: center of inversion; red: pseudo-center of inversion. Molecules of the same color are related by symmetry.

It may be noted that the molecular and packing changes in the **III** → **IV** transition are subtle. The conformations of the orcinol and bipyridine are largely the same as the corresponding molecular orientations along the unique axis (Fig. 5 ▶). The relationship between modulated structures has traditionally been understood in terms of relaxation of symmetry; a translation becomes a pseudo-translation and so on. In the context of the structural landscape, it may be suggested that this relaxed structure represents events that occur later in the reaction coordinate for crystallization. Table 2 ▶ shows that the low temperature form **IV** is more dense and better packed than form **III**. The relaxation of symmetry allows for a better packing and is in keeping with the idea of a landscape that is a profile of the energy events during crystallization. Fig. 6 ▶ shows the positions of the centers and pseudo-centers of inversion in form **IV** especially with respect to synthon **B**. In this figure, the symmetry designation of molecules is color coded.

In form **V** (*P*2_1_/*c*), the same type of hydrogen bonding (synthon **B**) is observed that is seen in forms **II** through to **IV**. The O—H⋯N hydrogen bonds are normal (1.76 Å, 176.1°; 1.76 Å, 179°; 1.78 Å, 178.5°; 1.78 Å, 175.2°; 1.79 Å, 173.4°; 1.80 Å, 174.8°; 1.80 Å, 173.3°; 1.81 Å, 169.4°). However, form **V** is different from forms **II** through to **IV** in that the ‘free’ bipyridine is missing. We suggest that synthon **B** can develop into structures **II** or **III** by picking up a free bipyridine, or that alternatively it can nucleate and grow as form **V** so that it is effectively a branch point in the landscape from which the crystallization events can proceed in two entirely different ways, depending on the experimental conditions.

The thermal profile of forms **II**, **III** and **V** in Fig. 4 ▶ shows that while form **II** has a clean single endotherm, the differential scanning calorimetry (DSC) of form **III** indicates some degree of conversion to form **II** and possibly the existence of form **IV** or some other uncharacterized form. Form **V** is in any case less stable and shows a broad endotherm lower than any of the other forms. Form **III** is accessible on the landscape and leads to other forms.

### 3.2. Supramolecular synthon based fragments approach (SBFA) for the compounds in this study and their relative stabilities   

The effectiveness of the SBFA method for transferability of multipole charge density parameters is due largely to the ability of the supramolecular synthon to act mechanistically as a modular unit. The electronic features of the synthon may be moved from one structure to another in the charge density analysis; such a procedure provides detail over and above what is obtained at the atomic and covalent bond level in the construction of ‘synthetic’ charge density maps, thereby giving a fine degree of agreement between theory and experiment (Hathwar, Thakur, Dubey *et al.*, 2011 ▶; Hathwar, Thakur, Row *et al.*, 2011 ▶).

In the context of the crystal landscape, the experimental crystal structures of the compound in question are generally mapped using the computational approach of Crystal Structure Prediction (CSP) (Sarma & Desiraju, 2002 ▶; Neumann *et al.*, 2008 ▶), in which possible crystal structures are predicted based on the energy–density profile. In spite of recent advances both in the algorithms as well as in increasing computational power, CSP of a multi-component system is still a challenge. Further, CSP protocol only takes into account the thermodynamic factors associated with packing, geometry optimization and clustering. It usually does not consider kinetic factors which are involved during the course of crystallization events. In order to fill this conceptual gap, experimental as well as theoretical charge density methods could be used (Koritsanszky & Coppens, 2001 ▶) which provide an energy profile of the immediate molecular vicinity because they address the system in terms of individual interactions. However, these rigorous methods have several hurdles such as the fact that very good crystals are needed, which diffract at high resolution (sinθ/λ ≥ 1.0), that the structures should be free from disorder and modulation, and that limitations like extinction and absorption should not be present. In the present study the multi-component orcinol–bipyridine system is both modulated and disordered and a rigorous charge density study is difficult. The utility of the transferable pseudo-atom databases approach in such situations is documented (Pichon-Pesme *et al.*, 1995 ▶; Domagala *et al.*, 2012 ▶; Dittrich *et al.*, 2006 ▶, 2013 ▶; Dominiak *et al.*, 2009 ▶; Volkov *et al.*, 2007 ▶); we have used the SBFA protocol which we have developed and which is well suited to this situation.

Forms **II** through to **V** were accordingly quantitatively rationalized with SBFA. Based on the structural description it is clear that the robustness and modular nature of hydrogen bonds associated with synthon **B** are the critical factors in applying SBFA to forms **II** through to **V**. This is quantified *via* multipole parameters of the O—H⋯N hydrogen bonds derived from high-resolution X-ray diffraction data. The transferability of the multipole parameters of the O—H⋯N hydrogen bonds in synthon **B**, and other interactions, in forms **II** through to **V**, generate charge density maps and provide the quantitative insights of electronic distribution in the intermolecular region through their topological parameters.

**Figure 7:**
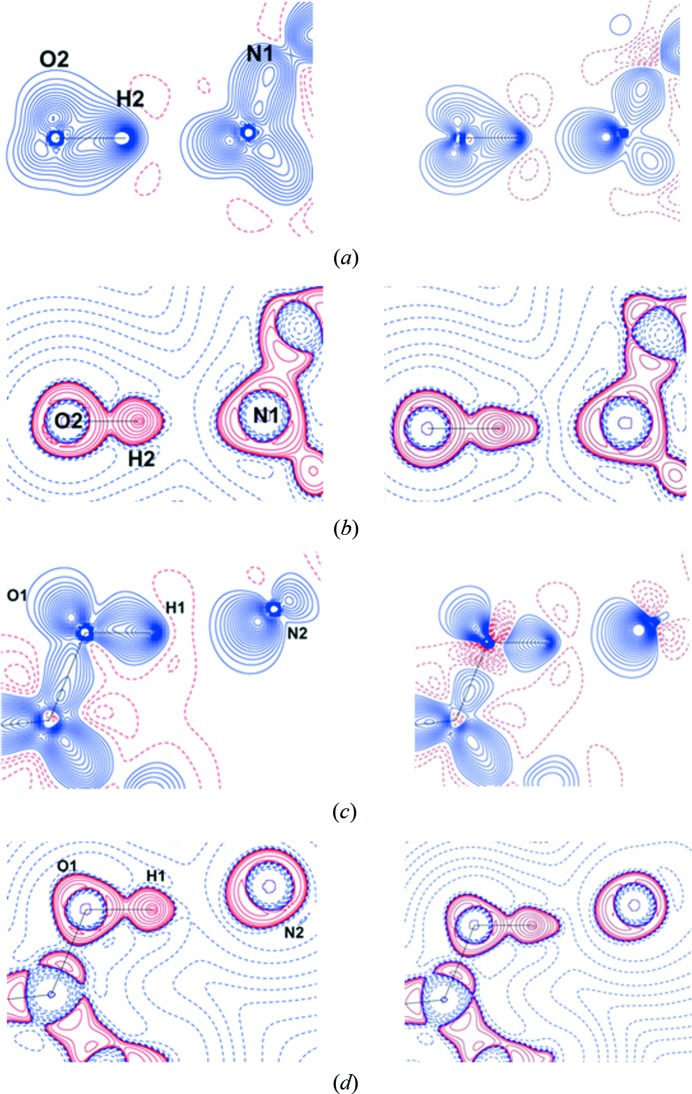
Comparison of deformation density and Laplacian maps [Δρ(*r*) and ∇^2^ρ(*r*)] maps, obtained from SBFA (left) and theoretical calculations based on *CRYSTAL09* (right), in O2—H2⋯N1 (*a*, *b*) and O1—H1⋯N2 (*c*, *d*) intermolecular space, respectively. Δρ(*r*) contours are drawn at ±0.05 e Å^−3^. ∇^2^ρ(*r*) (e Å^−5^) drawn in logarithmic scale.

The multi-component systems were divided, as described previously (Hathwar, Thakur, Dubey *et al.*, 2011 ▶), into logical fragments based on their synthons, which involve both strong and weak interactions (Fig. 1 ▶). Multipole parameters for the strong hydrogen bonds (O—H⋯N) present in all the structures were taken from the experimental data of the 4-hydroxybenzoic acid:isonicotinamide (4HBA:INA) cocrystal (Vishweshwar *et al.*, 2003 ▶), while the weaker ones C—H⋯O, C—H⋯N, C—H⋯π were chosen from an in-house library of synthons. The synthesized charge density features from SBFA were visualized through their deformation and Laplacian plots, which were in agreement with multipole refinements done on structure factors obtained from high-level density-functional theory calculations in *CRYSTAL09* (Dovesi *et al.*, 2009 ▶) (Fig. 7 ▶). The topological analysis of the intermolecular region was performed using the Quantum Theory of Atoms in Molecules (QTAIM), resulting in the location of bond-critical points between the strong as well as the weak interactions present in the crystal structures. The comparison was restricted to only forms **II** and **III** as our purpose was to verify the validity of the transferred model so that we could gain confidence in proceeding with the two other forms. In forms **IV** and **V**, which are more complex, the synthesized features were not compared with their theoretical values and were taken as they are. For form **I** we felt that the exercise itself was unfeasible because of the non-reproducibility of the form, as well as the slightly poor data quality of the already reported structure.

In form **II**, the topological analysis confirmed that O—H⋯N is the strongest interaction in the crystal structure. The remaining interactions present in the structure reflect their strengths in terms of their lower values of ρ and ∇^2^ρ. Comparison of the topological parameters between the SBFA model and theory deviates in only certain regions particularly for the strong O—H⋯N hydrogen bonds. The observed deviation in the Laplacian can be explained based on our previous work on the carboxylic dimer synthons, where it was attributed to the elongation of the O—H bonds. The comparable values between SBFA and theoretical topological parameters of covalent bonds and other weak intermolecular interactions support the validity of the transferred model (Table 3 ▶). The comparison of form **III** with theory and the topological parameters of forms **IV** and **V** have been summarized in the supporting information.

For the calculation of binding energies, it was also found convenient to define a molecular shell based on a coordination envelope around the asymmetric unit. In practice, all molecules that are found 0.2 Å beyond the van der Waals surface of the asymmetric unit (defined in terms of the closest group of bipyridine and orcinol molecules) constitute the shell. The shell consists of O—H⋯N hydrogen bonds, C—H⋯N and C—H⋯O hydrogen bonds and several weak C⋯C interactions. A critical-point search within the shell provides all the required contacts to be considered in the calculation. The Espinosa–Molins–Lecomte method (Espinosa *et al.*, 1998 ▶) was used to calculate the interaction energy (*E*_int_) using the Abramov expression (Abramov, 1997 ▶) which gives the kinetic and the potential energy density at the bond-critical points. The magnitude of the energy obtained by this approach is an indicator and not an absolute value, and hence a direct comparison with the values obtained from periodic DFT calculation may not be appropriate.

The binding energy, that is the energy of a molecular shell, was used to compute the relative stabilities of the different forms using the EML method (Nelyubina *et al.*, 2010 ▶). This was compared with the cohesive energy calculations performed using *CRYSTAL09*. In single-component crystals with *Z*′ = 1, the treatment of the cohesive energy is straightforward. When *Z*′ > 1 the computations are more involved but still manageable. In multi-component systems, however, the complexity of the calculations increases to a level that is unworkably tedious. In our system, the various forms do not even have the same *Z*′ values and one of them is also a pseudopolymorph. In such a scenario, the SBFA and EML methods provide a simplified method for calculation of energies, and this is a distinct advantage. The *CRYSTAL*09 and EML methods also have a slightly different physical interpretation which will be more clearly outlined in the next section.

The energies reported in Table 2 ▶ are obtained with *CRYSTAL09* and EML, where the energy *E*_coh_ corresponds to the energy of the defined asymmetric unit. The calculated energies cannot be used directly because the volume of the asymmetric unit is different in the five forms. Still, we attempted the quantification of forms **I** through to **IV** as they have the same 2:3 stoichiometric ratio. Even here we need to normalize the values because the number of molecules in the asymmetric unit is different in each of the forms. In this way, we find that form **I** is the least stable, and that forms **II** and **III** are equi­energetic. It was conjectured that the energy values are biased considerably by the complexity of the cocrystal formation and the values of *Z*′ and hence another calculation based on energy per molecule was performed.

### 3.3. Structural landscape   

The concept of the landscape follows naturally from the phenomena of polymorphism (Bernstein, 2002 ▶) and pseudopolymorphism and is conveniently applied to mono-component systems. In two-component systems, like the present case, it seems natural to assume that the earliest stages of recognition (smallest synthons) are heteromolecular in nature, for how else would a two-component system be obtained? The very fact that a two-component crystal **AB** is even obtained suggests that one or more interactions of the type **A**⋯**B** are better than any of the interactions of the type **A**⋯**A** or **B**⋯**B** (Sarma & Desiraju, 1985 ▶). In turn, these stable **A**⋯**B** heterosynthons permute themselves in different ways to give various polymorphs, so that one is, in effect, traversing the landscape. It may be supposed that the simple synthon **B** associates with other such synthons in solution without any symmetry constraints, and as crystallization becomes more enthalpy controlled, these clusters (aggregates, shell) gradually approach the final configurations as seen in forms **II** and **III** (more symmetry constraints), and forms **IV** and **V** (less symmetry constraints). In effect, the small synthons represent a certain ‘irreversible’ point and all subsequent events follow from it.

Structures **II** through to **V** may be understood as representing alternative arrangements of modules, that we have termed LSAMs. At this point, it is worth noting that the LSAM, as we have defined, has some similarity with the ‘growth unit’ as defined by Davey (Davey *et al.*, 2002 ▶) with the caveat that Davey‘s growth unit may also incorporate solvent. The similarity stems from the fact that all these species are pre-nucleation entities. The modularity of LSAMs permits an analysis of charge density data in terms of contributions from structural fragments that are treated in a similar way to the pseudo-atoms in the classical charge density transferability approaches. The modularity of these structural fragments also helps in their analysis from the viewpoint of the structural landscape. Modularity is the key link that connects all the structures that constitute the landscape.

**Figure 8:**
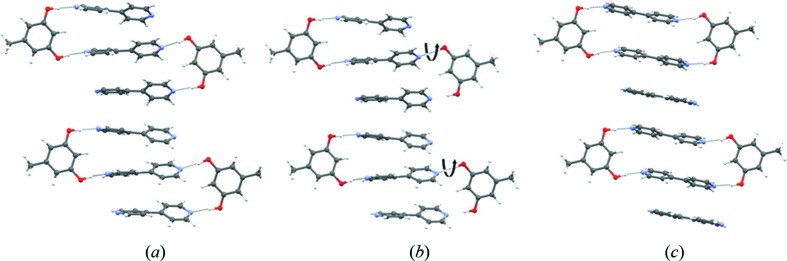
(*a*) Finite divergent arrangement (synthon **A**) of molecules in form **I**. (*b*) Possible rearrangement of molecules during the course of crystallization. (*c*) Finite convergent arrangement (synthon **B**) of molecules in forms **II** through to **V**.

**Figure 9:**
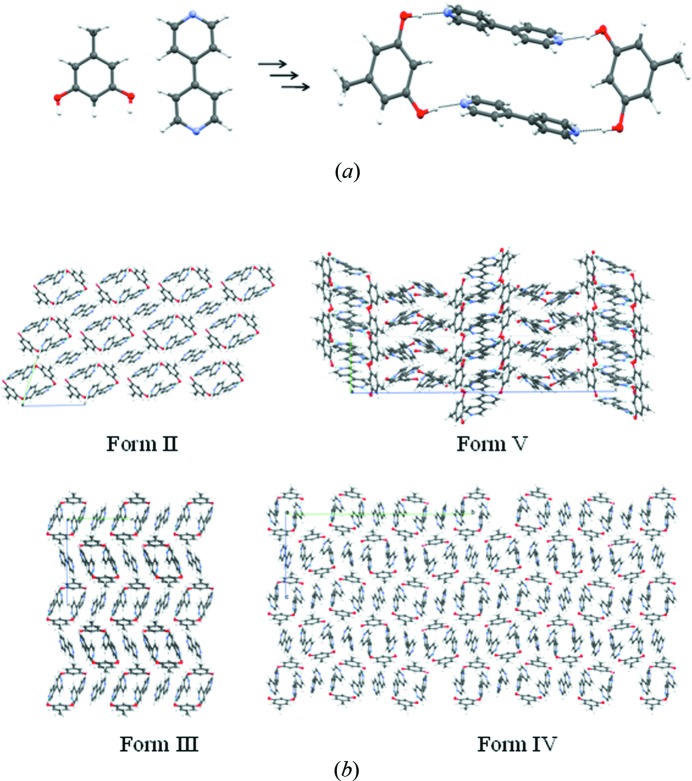
(*a*) Molecular recognition based on O—H⋯N hydrogen bonds and the formation of synthon **B**. (*b*) Various topologies of forms **II** through to **V**, anticlockwise, based on synthon **B**.

If there are two steps in the late stages of crystallization, finite strands of alternating bipyridine (three molecules) and orcinol (two molecules), which we have defined as synthon **A**, crystallize, in the first step, to give form **I** with O—H⋯N hydrogen bonds in the distance range 1.75–1.81 Å and an angle range 164–179°. This is shown in Fig. 8 ▶(*a*). It is not at all difficult to conceive a process in which successive O—H⋯N hydrogen bonds are made and broken (Fig. 8 ▶ *b*) and orcinol molecules rotate nearly 180° so that the structure evolves into that of form **II** with its closed synthon (Fig. 8 ▶ *c*). In this scenario, form **I** would be a kinetic form. Although not proof, the fact that we were unable to obtain form **I** again would hint that it is also metastable. To summarize, there are two aggregation possibilities of the LSAMs. In both possibilities the third bipyridine is an active participant either being hydrogen bonded (form **I**) or facilitating close packing (forms **II** through to **IV**). Form **V** is different and develops independently from synthon **B** not requiring the free bipyridine. This is shown in Fig. 9 ▶.

During navigation of the nucleation pathway of forms **II** through to **IV**, the system exploits the modular nature of synthon **B** as a means of achieving the structures based on their energies. As we have already discussed, forms **II** and **III** are similar at the smaller aggregate, and this suggests that up to a certain point the modular unit follows the same nucleation path, but as the aggregation level becomes larger and larger there are shifts leading to a choice between two different pathways – finally this results in two different crystal structures. The quantitative analysis leading to the estimates of interaction energies using two different approaches (*CRYSTAL09* and SBFA/EML) brings out the significance of the regions of the landscape between forms **II** and **III**. In practical terms, both forms appear to be practically equivalent in that they lie in nearly the same region of the energy–density plot. On the other hand, form **II** shows slightly better packing than form **III**, and after the phase transition, form **III** converts into form **IV** which is packed as efficiently as form **II** (Table 2 ▶). This emergence of better packing is the result of the relaxation in the symmetry constraints in form **III**. In the context of the landscape, such behaviour is interesting. Forms **II** and **IV** have nearly the same densities, energies and packing efficiencies but they represent entirely different pathways in the landscape, and their structures are also quite different. The relative stabilities of forms **II**, **III** and **IV** with similar asymmetric units are selected below.
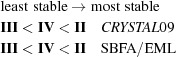



It is unlikely that forms **II** and **IV** can interconvert easily, but they have the lowest energies among the forms isolated and studied here. Which is the global minimum? Is there another, yet undiscovered form, which is of even lower energy? If not, is it fair to speak of two independent crystallization pathways, each leading to a stable outcome? In this case, how relevant were early versions of the CSP blind tests that demanded only the top three choices for a molecule or a cocrystal? Information, such as is obtained here, regarding various possibilities of nucleation pathways in the reaction coordinate during the course of crystallization, such as the existence of form **III**, and which finally leads to form **IV**, which seems to be just as favourable as form **II**, conveys that there need not be just one structure at the global minimum. More than ever, there is now a compelling feeling that the concept of ‘crystal structure’ is not unique. The comment needs emphasis: rather than speak of *the crystal structure* of a molecule, a term that may have only a limited meaning in the landscape context, it may be fairer to speak of *a crystal structure* of a molecule (in this case a molecular system, because we are dealing with cocrystals). A particular structure is just one of many.

During the solution → crystal pathway, there could be various metastable forms and structural fragments which may not appear in the final *crystal structure*. These metastable structures are the outcome of crystal synthesis because of the competition between kinetic and thermodynamic factors associated with the transition from an entropy dominated scenario in solution to an enthalpically determined crystal. These (metastable) crystal structures (known as polymorphs or pseudopolymorphs) which encapsulate thermodynamic factors as well as energy–density profiling constitute a large landscape which may be accessed either by the hydrogen-bond hierarchies during the solution to the supersaturated state or *via* thermal transformations in the final stages of crystallization. In solution, molecules recognize one another based on their complementary hydrogen-bonding functionalities which define the basic kinetic units of the crystal structure, namely supramolecular synthons. Even as the hydrogen-bond hierarchies are established, and based on their respective energies, synthons optimize themselves right from the initial to intermediate to final stages of crystallization until chemically (kinetic) or geometrically (thermodynamic) reasonable structures are obtained for the organic compound in question. This is the final stage of crystallization in which the dichotomies between synthon *versus* close packing become fully manifest. Alternatively, one might reduce the molecule → crystal progression in the landscape into a discussion of packing (thermodynamic aspects) and synthon theory (kinetic aspects). We would urge an appreciation of both these viewpoints in the current scenario (Kitaigorodskii, 1973 ▶; Dunitz & Gavezzotti, 2005 ▶; Desiraju, 2007 ▶).

## 4. Conclusions   

This work addresses several questions that highlight the difficulties faced in dealing with complex systems in crystal engineering. Our approaches have posed questions that future methodologies should hopefully address. We have shown that the closed zero-dimensional convergent phenol⋯pyridine synthon (synthon **B**) is robust and constitutes the element of modularity that causes extensive polymorphism in the orcinol−bipyridine system. Within the context of the present example, it is therefore not possible to state that cocrystal formation decreases the likelihood of polymorphism. It is true that the major interaction, namely O—H⋯N, is conserved in all the forms but the polymorphism is caused by a variation in the more minor interactions, in other words in the ways in which synthon **B** modules are arranged with respect to one another. Larger assemblies of synthon **B** may be termed as *Long Range Synthon Aufbau Modules* or LSAM. This work also shows that this collection of polymorphs of orcinol−bipyridine constitutes a landscape which may be studied by an energy profiling and interaction profiling, both of which may be carried out with charge density studies, more particularly with our newly suggested technique of *Supramolecular Synthon Based Fragments Approach* (SBFA) for transferability of multipole parameters. The synthon is a modular structural unit that lends itself particularly well to the transferability of electron density information in a crystal structure. The polymorphs of orcinol−bipyridine are structurally complex in a manner that renders them problematic for other methods of charge density analysis and our method offers some choice in this regard. The idea of a structural landscape that is defined by polymorphs, solvates and computed structures provides an indication about events in the late stages of crystallization. While computational CSP inputs on the thermodynamics of these events, or vertical profiling of the landscape, charge density methods give information on the interactions themselves and therefore, in principle, can lead to horizontal profiling and a measure of the kinetics that underlie crystallization events because in the end it is the energy and distance dependence of individual interactions that determine actual crystallization pathways which are essentially kinetically governed.

## Supplementary Material

Crystal structure: contains datablock(s) global, II, III_120K, III_140K, III_160K, III_200K, III_296K, IV, V. DOI: 10.1107/S2052252513024421/lc5058sup1.cif

Structure factors: contains datablock(s) II. DOI: 10.1107/S2052252513024421/lc5058IIsup2.fcf

Structure factors: contains datablock(s) III_120K. DOI: 10.1107/S2052252513024421/lc5058III_120Ksup3.fcf

Structure factors: contains datablock(s) III_140K. DOI: 10.1107/S2052252513024421/lc5058III_140Ksup4.fcf

Structure factors: contains datablock(s) III_160K. DOI: 10.1107/S2052252513024421/lc5058III_160Ksup5.fcf

Structure factors: contains datablock(s) III_200K. DOI: 10.1107/S2052252513024421/lc5058III_200Ksup6.fcf

Structure factors: contains datablock(s) III_298K. DOI: 10.1107/S2052252513024421/lc5058III_298Ksup7.fcf

Structure factors: contains datablock(s) IV. DOI: 10.1107/S2052252513024421/lc5058IVsup8.fcf

Structure factors: contains datablock(s) V. DOI: 10.1107/S2052252513024421/lc5058Vsup9.fcf

Additional supporting information. DOI: 10.1107/S2052252513024421/lc5058sup10.pdf

CCDC references: 944960, 944961, 944962, 944963

## Figures and Tables

**Table 1:** Crystallographic details of forms **II** through to **V**

	**II**	**III**	**IV**	**V**
CCDC No.	944960	944963	944961	944962
Stoichiometric ratio (taken)	2:3	1:2	1:2	2:1
Stoichiometric ratio (in the crystal)	2:3	2:3	6:9	4:4
Method of crystallization	Solvent evaporation	Melt sublimation	From polymorph **III** at around 150 K	Solvent evaporation
Solvent	CH_3_NO_2_	–	–	CH_3_NO_2_
Crystal color	Light yellow	Colorless	Colorless	Yellow
Melting point (K)	453.2	453.4	–	448.9
Molecular formula	C_7_H_8_O_2_·C_15_H_12_N_3_	C_7_H_8_O_2_·C_15_H_12_N_3_	C_21_H_24_O_6_·C_45_H_36_N_10_	C_28_H_32_O_8_·C_40_H_32_N_8_
Formula weight	358.42	358.42	1075.26	1121.31
Crystal system	Triclinic	Monoclinic	Monoclinic	Monoclinic
Space group	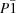	*P*2_1_/*n*	*P*2_1_/*n*	*P*2_1_/*c*
*a* (Å)	8.7711 (6)	9.0828 (3)	9.2118 (2)	17.8201 (5)
*b* (Å)	10.011 (1)	12.3446 (4)	36.2075 (7)	8.3288 (2)
*c* (Å)	12.0057 (9)	16.6095 (4)	16.5458 (4)	39.3222 (9)
α (°)	67.978 (8)	90	90	90
β (°)	78.030 (6)	96.320 (2)	97.923 (2)	91.901 (4)
γ (°)	69.224 (8)	90	90	90
*V* (Å^3^)	910.4 (1)	1851.0 (1)	5465.9 (2)	5833.0 (3)
ρ_calc_ (g cm^−3^)	1.307	1.286	1.307	1.277
*F*(000)	378	756	2268	2368
μ (mm^−1^)	0.086	0.084	0.085	0.085
*T* (K)	100 (2)	160 (2)	100 (2)	100 (2)
λ (Å)	0.71073	0.71073	0.71073	0.71073
Reflections collected	16 503	12 420	49 748	39 460
Unique reflections	3998	4066	11 990	12 764
Completeness (%)	99.9	100	99.9	99.9
Redundancy	4.0	3.1	4.1	3.1
*R*_int_	0.035	0.029	0.050	0.075
*R*_1_ (*F*)	0.039	0.052	0.056	0.073
*wR*_2_ (*F* ^2^)	0.102	0.149	0.121	0.196
Goodness-of-fit	1.042	1.060	1.013	1.024
2θ_max_	54	54	54	54

**Table 2:** KPI, crystal densities and energies for the compounds in this study

				Normalized†		Per molecule energy‡	
	KPI (%)	Calculated crystal density (g cm^−3^)	Energy *CRYSTAL09* (kJ mol^−1^)	*CRYSTAL09* (kJ mol^−1^)	EML (kJ mol^−1^)	*CRYSTAL09* (kJ mol^−1^)	EML (kJ mol^−1^)
Form **I** §	69.9	1.305	−684.88	−684.88	−	−143.89	−
Form **II**	70.7	1.308	−567.74	−1135.48	−957.0	−155.10	−153.46
Form **III** §	69.3	1.286	−634.77	−1269.54	−716.6	−141.54	−130.45
Form **IV**	70.6	1.307	−1888.43	−1259.58	−697.4	−144.18	−139.54
Form **V**	68.9	1.282	−925.59	–	–	−144.64	–

† Normalized: energy as a multiple of the (2:3) asymmetric unit.

‡ The calculation has been described in the supporting information.

§ Data collection at 150 K.

**Table 3:** Numerical (top) and graphical (bottom) comparison of the topological parameters, SBFA and theory (italics), of form **II**

Synthon	ρ (e Å^−3^)	∇^2^ρ (e Å^−5^)	*R*_ij_ (Å)	∊	*G* (kJ mol^−1^ bohr^−3^)	*V*(kJ mol^−1^ bohr^−3^)	|*V*|/*G*
O2—H2⋯N1	0.34	2.5	1.738	0.05	97.24	−127.61	1.31
	*0.41*	*0.8*	*1.734*	*0.03*	*84.77*	−*147.80*	*1.74*
O1—H1⋯N2	0.31	2.4	1.7663	0.06	85.87	−107.93	1.26
	*0.36*	*1.5*	*1.7666*	*0.04*	*83.58*	−*125.43*	*1.50*
C9—H9⋯O1	0.05	0.8	2.5076	0.21	16.64	−10.98	0.66
	*0.05*	*0.7*	*2.4816*	*0.19*	*15.02*	*−10.68*	*0.71*
C20—H20⋯O2	0.03	0.6	2.6806	0.44	11.30	−7.16	0.63
	*0.03*	*0.6*	*2.6167*	*0.21*	*12.12*	*−7.98*	*0.66*
C11—H11⋯C6	0.04	0.8	2.5472	0.97	16.42	−10.58	0.64
	*0.08*	*0.5*	*2.5301*	*0.08*	*14.02*	*−13.24*	*0.94*
C4—H4⋯N1	0.04	0.5	2.7078	0.44	9.45	−6.65	0.70
	*0.04*	*0.5*	*2.6603*	*0.11*	*14.71*	*−9.92*	*0.67*
C8—H8⋯N3	0.03	0.6	2.6795	1.18	12.15	−7.66	0.63
	*0.03*	*0.6*	*2.633*	*0.01*	*11.66*	*−8.69*	*0.75*
