# Supplementary material for: Crystal landscape in the orcinol:4,4′-bipyridine system: synthon modularity, polymorphism and transferability of multipole charge density parameters
Source: IUCrJ. 2013 Oct 1;1(Pt 1):8–18. doi: 10.1107/S2052252513024421 (PMC4104971; doi:10.1107/S2052252513024421)
Supplement: Supplementary file 10 [file m-01-00008-sup10.pdf]

Supplementary Information

Crystal Landscape in the Orcinol:4,4′-Bipyridine System: Synthon Modularity, Polymorphism and Transferability of Multipole Charge Density Parameters

Ritesh Dubey, Mysore S. Pavan, Tayur N. Guru Row*, Gautam R. Desiraju*
Solid State and Structural Chemistry Unit, Indian Institute of Science,
Bangalore 560 012, India
ssctng@sscu.iisc.ernet.in; desiraju@sscu.iisc.ernet.in

TABLES

Table 1: Crystallographic Table for variable temperature data sets of Form **III**
Table 2: Comparison of the topological parameters in SBFA and Theory of Form **III**
Table 3: Intermolecular interaction table of the topological parameters of Form **IV**
Table 4: Intermolecular interaction table of the topological parameters of Form **V**
Table 5: High resolution experimental data of 4-hydroxybenzoic acid:Isonicotinamide
Table 6: Intermolecular interaction table of the topological parameters of 4-hydroxybenzoic acid:Isonicotinamide
Table 7: Topological parameters of the intramolecular bonding region in 4-hydroxybenzoic acid:Isonicotinamide
Table 8: Comparisons of the *bcp* properties for intramolecular bonding region for Form **II**
Table 9: Comparisons of the *bcp* properties for intramolecular bonding region for Form **III**
Table 10: Topological parameters of the intramolecular bonding region in Form **IV** obtained from SBFA
Table 11: Topological parameters of the intramolecular bonding region in Form **V** obtained from SBFA

SCHEME

Scheme 1: Supramolecular synthons and the corresponding in-house SBFA library entry

FIGURES

Figure 1: ORTEP diagrams for the asymmetric unit of the crystal structures (ellipsoids are drawn at 50% probability)
Figure 2: Packing diagrams for Forms **I** through **V**
Figure 3: Variation of U_{ij} with temperature in Form **III**
Figure 4: Scatter plot depicting the variation of Fobs with Fcal for **4HYINA** (left) Variation of F_{obs}/F_{cal} with $(\sin\theta)/\lambda$ (right)
Figure 5: Experimental residual density, deformation density and Laplacian maps of 4-hydroxybenzoic acid:Isonicotinamide
Figure 6: Deformation density and Laplacian maps of intermolecular O–H···N synthon in 4-hydroxybenzoic acid:Isonicotinamide
Figure 7: Deformation and Laplacian diagrams of Form **II** (SBFA and Theory)
Figure 8: Deformation and Laplacian diagrams of Form **III** (SBFA and Theory)
Figure 9: Deformation and Laplacian diagrams of Form **IV** from SBFA
Figure 10: Deformation density and Laplacian maps of Form **V** from SBFA.

Table 1: Crystallographic Table for Variable Temperature Data Sets of Form **III**

**Orcinol:4,4′-Bipyridine (Form III)**					
CCDC No.	944964	944965	944963	944966	944967
Crystal colour	Colourless	Colourless	Colourless	Colourless	Colourless
Molecular formula	$C_{14}H_{16}O_4.C_{30}H_{24}N_6$	$C_{14}H_{16}O_4.C_{30}H_{24}N_6$	$C_{14}H_{16}O_4.C_{30}H_{24}N_6$	$C_{14}H_{16}O_4.C_{30}H_{24}N_6$	$C_{14}H_{16}O_4.C_{30}H_{24}N_6$
Formula weight	716.83	716.83	716.83	716.83	716.83
Crystal system	Monoclinic	Monoclinic	Monoclinic	Monoclinic	Monoclinic
Space group	$P2_1$/n	$P2_1$/n	$P2_1/n$	$P2_1$/n	$P2_1$/n
a (Å)	9.1938(4)	9.1180(3)	9.0828(3)	9.2233(3)	9.2233(3)
b (Å)	12.3828(6)	12.3427(4)	12.3446(4)	36.2938(12)	36.2938(12)
c (Å)	16.7311(8)	16.6400(4)	16.6095(4)	16.5853(5)	16.5853(5)
α (˚)	90	90	90	90	90
β (˚)	96.358(4)	96.381(3)	96.320(2)	97.827(3)	97.827(3)
γ (˚)	90	90	90	90	90
V ($Å^3$)	1893.04(15)	1861.08(10)	1851.0(1)	5500.2(3)	5500.2(3)
Z	4	4	4	4	4
ρ_{calc} (g/cm^3)	1.258	1.279	1.286	1.298	1.298
F(000)	756	756	756	2268	2268
μ. (mm^{-1})	0.082	0.084	0.084	0.085	0.085
T (K)	**296(2)**	**200(2)**	**160(2)**	**140(2)**	**120(2)**
λ (Å)	0.71073	0.71073	0.71073	0.71073	0.71073
Reflns. collected	13235	12473	12420	38087	38087
Unique reflns.	4129	4065	4066	12593	12593
Completeness (%)	99.9	100	100	99.6	99.6
R_{int}	0.048	0.028	0.029	0.051	0.051
R_1 (F)	0.0673	0.0583	0.052	0.0672	0.0672
wR_2 (F^2)	0.2324	0.1699	0.149	0.1643	0.1642
Goodness-of-fit	1.064	1.083	1.060	1.034	1.034
$2\theta_{max}$	54	54	54	54	54

Table 2: Comparison of the intermolecular topological parameters, in SBFA and Theory of Form **III**

Synthon	ρ(eÅ^{-3})	$\nabla^2\rho$(eÅ^{-5})	R_{ij}(Å)	ε	G(kJmol^{-1}bohr^{-3})	V(kJmol^{-1}bohr^{-3})	|V|/G
O1−H1···N1	0.31	2.4	1.7946	0.05	87.85	–110.34	1.26
CRYSTAL09	0.34	2.4	1.7520	0.02	95.22	–125.08	1.31
O2−H2···N2	0.31	2.3	1.7478	0.05	86.04	–109.43	1.27
CRYSTAL09	0.34	2.7	1.7575	0.01	93.30	–113.06	1.21
C15−H15···O2	0.04	0.6	2.6673	0.34	12.35	–8.36	0.68
CRYSTAL09	0.06	0.8	2.5712	0.36	17.39	–13.0	0.75
C9−H9···O1	0.03	0.6	2.6258	0.68	11.79	–7.25	0.61
CRYSTAL09	0.06	0.8	2.5464	0.14	17.39	–13.0	0.75

Table 3: Intermolecular interaction table of the topological parameters of Form **IV**

Synthon	ρ(eÅ^{-3})	$\nabla^2\rho$(eÅ^{-5})	R_{ij}(Å)	ε	G(kJmol^{-1}bohr^{-3})	V(kJmol^{-1}bohr^{-3})	|V|/G
O5–H5O···N1	0.31	2.3	1.7608	0.04	85.03	–107.51	1.26
O4–H4O···N2	0.3	2.2	1.7702	0.05	82.41	–104.09	1.26
O6–H6O···N3	0.33	2.4	1.742	0.03	91.74	–118.46	1.29
O3–H3O···N4	0.31	2.4	1.7514	0.04	87.22	–110.38	1.27
O1–H1O···N8	0.30	2.2	1.7708	0.04	83.36	–105.97	1.27
O2–H2O···N9	0.32	2.4	1.7406	0.04	90.58	–116.05	1.28
C60–H60···O6	0.04	0.7	2.6558	0.11	14.03	–9.48	0.68
C50–H50A···O2	0.03	0.6	2.6383	0.14	11.23	–7.28	0.65
C52–H52···O2	0.04	0.8	2.5687	0.47	14.97	–9.23	0.62
C11–H11C···O3	0.04	0.6	2.6425	0.04	11.77	–7.64	0.65
C15–H15···O3	0.02	0.5	2.7555	0.86	9.59	–5.73	0.6
C24–H24···O3	0.05	0.8	2.6373	0.05	15.84	–11.09	0.7
C54–H54···O4	0.02	0.6	2.6649	0.47	11.5	–6.68	0.58
C47–H47···O4	0.06	1.0	2.4547	0.16	19.96	–13.7	0.69
C33–H33A···O5	0.03	0.5	2.6857	0.11	9.98	–6.34	0.64
C34–H34···O5	0.03	0.6	2.6901	0.79	11.21	–6.81	0.61
C2–H2···O5	0.05	0.9	2.5278	0.24	18.13	–12.67	0.7
C17–H17···O6	0.02	0.5	2.7215	0.87	9.86	–5.46	0.55
C13–H13···N8	0.05	0.5	2.9731	1.02	12.01	–9.18	0.76
C13–H13···N9	0.04	0.5	2.6921	0.23	10.03	–6.76	0.67
C38–H38···N2	0.03	0.5	2.7982	0.78	9.39	–6.2	0.66
C47–H47···N2	0.03	0.4	3.3672	0.16	8.05	–5.23	0.65
C56–H56···N3	0.04	0.5	2.7735	1.84	10.68	–7.18	0.67
C11–H11B···N5	0.05	0.7	2.7709	0.21	14.34	–10.03	0.7

C44–H44···N6	0.05	0.8	2.5793	0.58	16.23	–10.98	0.68
C63–H63···N7	0.04	0.9	2.4907	0.24	17.32	–10.67	0.62

Table 4: Intermolecular interaction table of the topological parameters of form **V**

Synthon	ρ(eÅ^{-3})	$\nabla^2\rho$(eÅ^{-5})	R_{ij}(Å)	ε	G(kJmol^{-1}bohr^{-3})	V(kJmol^{-1}bohr^{-3})	|V|/G
O5–H5O···N1	0.30	2.2	1.788	0.04	82.53	–104.50	1.27
O3–H3O···N2	0.32	2.4	1.755	0.04	90.07	–115.79	1.29
O6–H6O···N3	0.30	2.2	1.795	0.04	80.24	–101.26	1.26
O4–H4O···N4	0.28	2.2	1.8105	0.02	77.55	–96.17	1.24
O2–H2O···N5	0.31	2.3	1.764	0.04	86.35	–110.30	1.28
O7–H7O···N7	0.30	2.2	1.783	0.04	83.36	–105.97	1.27
O8–H8O···N8	0.30	2.2	1.7769	0.04	82.30	–104.03	1.26
O1–H1O···N6	0.29	2.2	1.7979	0.03	79.51	–99.87	1.26
C7–H7···O2	0.06	0.8	2.5673	0.12	17.44	–12.55	0.72

Cohesive Energy calculations:

Energy computations were performed with the *CRYSTAL09* program package (Dovesi *et al.*, 2009) at the DFT (B3LYP) level of theory, employing the 6-31G** basis set. Both Grimme dispersion correction and correction for basis set superposition error (BSSE) were applied. Ghost atoms were selected up to 4 Å distance from the considered molecule in a crystal lattice, and were used for the BSSE estimation.

The cohesive energy (E_{coh}) was calculated using the formula:

$$E_{coh} = \frac{1}{Z} E_{bulk} - E_{mol}$$

where E_{bulk} is the total energy of a system (calculated per unit cell) and E_{mol} is the energy of a molecule extracted from the bulk . Z stands for the number of molecules in the unit cell. In the present case two approaches were tested. First, each molecule was considered separately. Then, the E_{mol} was calculated for all the molecules in the asymmetric unit and added. In the second approach, the energy of all the molecules in the asymmetric unit was calculated together. In both the cases the molecular fragment energy was calculated with BSSE correction. In all the calculations the X–H bonds were normalized to neutron values. In the end the second approach is the one reported because the asymmetric unit consists of a complex cluster of more than two (different) molecules and their combined energy offers a more meaningful description of E_{mol} in the present scenario.

In addition to the above method we also attempted to quantify the cohesive energies to a per-molecule description which is more appealing. In the per-molecule treatment the term E_{mol} was obtained by averaging the molecular energies of bypyridyl and orcinol's separately and later they

were averaged according to their ratios in the different forms. The value of Z in this method was taken as total number of molecules in the unit cell compared to the total number of molecular clusters in the previous method. These values were compared with the EML method also by a similar treatment where the per-molecule treatment was with respect to the asymmetric unit.

Table 5: High resolution experimental data of 4-hydroxybenzoic acid:Isonicotinamide (**4HYINA**)

CCDC No.	944959	***λ (Å)***	0.71073
Molecular formula	$C_{13}\ H_{12}\ N_2\ O_4$	***Reflns. collected***	169020
Formula weight	260.25	***Unique reflns.***	12238
Crystal system	Monoclinic	***Completeness (%)***	100
Space group	$P2_1/n$	***Redundancy***	13.8
a (Å)	6.0667(4)	$\boldsymbol{R_{int}}$	0.037
b (Å)	9.3620(6)	***Spherical atom refinement***	
c (Å)	20.525(1)	$\boldsymbol{R_1\ (F)}$	0.0398
α (°)	90	$\boldsymbol{wR_2\ (F^2)}$	0.1174
β (°)	95.033(3)	***Goodness-of-fit***	1.048
γ (°)	90	***Multipole refinement***	
$\boldsymbol{V\ (Å^3)}$	1161.3(1)	***Reflns. used*** **[*I* > 3σ(*I*)]**	9494
Z	4	***No of parameters***	359
$\boldsymbol{\rho_{calc}\ (gcm^{-3})}$	1.489	$\boldsymbol{R_1\ (F^2)}$	0.021
F(000)	544	$\boldsymbol{wR_2\ (F^2)}$	0.053
$\boldsymbol{\mu\ (mm^{-1})}$	0.112	***Goodness-of-fit***	1.211
T (K)	100(2)	$\boldsymbol{\Delta\rho_{min},\ \Delta\rho_{max}\ (eÅ^{-3})}$	–0.194, 0.206

Multipole Modeling: The charge density modeling and multipolar aspherical atom refinements were performed based on the Hansen and Coppens multipole formalism using XD2006 (Hansen & Coppens, 1978; Volkov *et al.*, 2006). The function, $\Sigma w\ (|F_o|^2 - K|F_c|^2)^2$ was minimized for all reflections with I >3σ (I). Weights (w) were taken as $1/\sigma^2\ (F_o^2)$ and convergence criterion of the refinement was set to a maximal shift/esd $<10^{-10}$. Su-Coppens-Macchi wave functions (Su & Coppens, 1998; Macchi & Coppens, 2001) were used for the core and valence scattering factors of all the atoms. The scale factor was refined against the whole resolution range of diffraction data in the first refinement step. The scatter plot of the variation of F_{obs} with F_{cal} is indicative of the quality of the data set after scaling. (Figure 2) The positional and anisotropic displacement parameters of the non-hydrogen atoms were refined using reflection data with sin $\theta/\lambda > 0.7$ Å^{-1}. In the next step of refinement, the position and displacement parameters of the non-hydrogen atoms were fixed to the refined values. The C—H bond length was constrained to the values obtained from neutron diffraction experiments reported in literature. The isotropic displacement parameters of the H-atom was refined initially with reflection data sin $\theta/\lambda < 0.7$ Å^{-1}. Further, the converged model was used to calculate anisotropic displacement parameters of H-atom using the

SHADE2 analysis (Madsen, 2006; Munshi *et al.*, 2008). ADP value of the H-atom obtained from SHADE2 analysis was kept fixed during the subsequent multipole refinements. Further scale, positional and anisotropic displacement parameters, P_{val}, P_{lm}, κ and κ′on non-hydrogen atoms were refined in a stepwise manner, until the convergence criterion was reached. Separate κ and κ′ were used to define different non-H atom type based on their chemical environments. For the hydrogen atoms the value was fixed at 1.2. The multipole expansion was carried out upto the octupole level for non-hydrogen atoms (l = 3). For the H atom, only monopole, bond directed dipole (d_z) and quadrupole (q_{3z^2-1}) components were refined during the multipole refinements. The quantitative analysis of the electron density topology and related properties was performed using the XDPROP module of XD software suite. Crystallographic refinement details of both spherical and multipolar model are summarized in Table 5.

Table 6: Intermolecular interaction table of the topological parameters of 4-hydroxybenzoic acid:Isonicotinamide

Interaction	ρ_{bcp}	$\nabla^2\rho_{bcp}$	R_{ij}	d_1	d_2	λ_1	λ_2	λ_3	ε
O3–H4⋯N1*	0.28	2.2	1.7745	1.1932	0.5813	–1.98	–1.91	6.14	0.04
O1–H1⋯O4	0.38	2.3	1.5688	1.0859	0.4829	–3.33	–3.23	8.84	0.03
N2–H2B⋯O2	0.13	1.9	1.9790	1.2779	0.7011	–0.76	–0.74	3.37	0.02
N2–H2A⋯O2	0.03	0.6	2.5573	1.5089	1.0484	–0.16	–0.10	0.90	0.67
C11–H11⋯O2	0.03	0.7	2.5522	1.5312	1.0210	–0.13	–0.10	0.92	0.29
N2–H2A⋯O1	0.05	0.8	2.5318	1.4593	1.0724	–0.21	–0.17	1.20	0.21
C8–H8⋯O1	0.06	1.0	2.4745	1.4293	1.0452	–0.22	–0.20	1.40	0.06

*This interaction was the most important for the present study

Table 7: Topological parameters of the intramolecular bonding region in 4-hydroxybenzoic acid:Isonicotinamide

Bond	ρ	$\nabla^2\rho$	R_{ij}	d_1	d_2	λ_1	λ_2	λ_3	ε
O1–C7	2.39	–21.5	1.3241	0.7937	0.5303	–21.3	–18.8	18.6	0.13
O1–H1	2.00	–42.5	1.0180	0.8072	0.2108	–35.3	–35.0	27.8	0.01
O2–C7	3.02	–35.5	1.2277	0.7693	0.4584	–30.3	–25.4	20.2	0.19
O3–C4	2.18	–15.4	1.3501	0.7897	0.5604	–18.9	–16.8	20.3	0.12
O3–H4	2.23	–37.0	0.9921	0.7665	0.2255	–36.1	–35.2	34.3	0.03
O4–C13	2.85	–29.5	1.2437	0.7449	0.4988	–26.5	–23.0	19.9	0.15
N1–C8	2.38	–18.7	1.3385	0.7525	0.5860	–19.5	–17.3	18.1	0.13
N1–C12	2.45	–20.1	1.3402	0.7530	0.5872	–21.2	–17.4	18.5	0.22
N2–C13	2.45	–22.3	1.3311	0.7746	0.5565	–22.1	–17.5	17.3	0.26

N2–H2A	1.92	–29.6	1.0101	0.7886	0.2214	–28.4	–27.3	26.1	0.04
N2–H2B	2.20	–34.8	1.0100	0.7659	0.2442	–32.1	–30.7	27.9	0.04
C1–C2	2.18	–17.3	1.4002	0.6860	0.7142	–17.6	–14.4	14.8	0.22
C1–C6	2.12	–16.1	1.3981	0.7026	0.6955	–16.9	–13.8	14.6	0.22
C1–C7	1.84	–12.2	1.4773	0.7105	0.7668	–14.7	–12.0	14.5	0.22
C2–C3	2.18	–17.9	1.3858	0.7047	0.6810	–17.8	–13.9	13.8	0.28
C2–H2	1.75	–14.5	1.0831	0.7431	0.3399	–17.7	–16.4	19.5	0.08
C3–C4	2.17	–18.0	1.4004	0.6930	0.7074	–18.1	–14.2	14.3	0.27
C3–H3	1.92	–19.8	1.0831	0.7031	0.3800	–18.9	–18.0	17.1	0.05
C4–C5	2.21	–18.2	1.4013	0.7174	0.6839	–18.3	–14.4	14.5	0.27
C5–C6	2.15	–17.1	1.3874	0.6883	0.6991	–17.2	–13.8	13.9	0.25
C5–H5	1.79	–14.5	1.0830	0.7388	0.3442	–17.7	–16.6	19.7	0.07
C6–H6	1.86	–17.0	1.0830	0.7167	0.3663	–18.4	–17.3	18.7	0.07
C8–C9	2.23	–19.3	1.3907	0.7094	0.6812	–18.3	–14.7	13.8	0.24
C8–H8	1.92	–19.0	1.0834	0.7158	0.3677	–19.4	–18.0	18.4	0.08
C9–C10	2.17	–17.4	1.3922	0.6931	0.6991	–17.8	–13.9	14.3	0.28
C9–H9	1.75	–16.7	1.0830	0.7394	0.3436	–18.0	–16.7	18.0	0.08
C10–C11	2.13	–17.1	1.3914	0.6894	0.7020	–17.0	–14.2	14.0	0.19
C10–C13	1.79	–11.6	1.4968	0.7445	0.7523	–14.3	–11.9	14.6	0.2
C11–C12	2.23	–18.6	1.3925	0.6687	0.7238	–18.4	–14.2	14.0	0.29
C11–H11	1.75	–16.8	1.0834	0.7499	0.3334	–17.8	–17.3	18.3	0.03
C12–H12	1.68	–16.1	1.0832	0.7463	0.3369	–17.6	–16.1	17.5	0.09

Table 8: Numerical (top) and graphical (bottom) comparison of the *bcp* properties for intramolecular bonding region obtained from SBFA modeled data with that obtained from theoretical calculations performed at B3LYP/6-31G(d,p) level of theory (CRYSTAL09) for Form **II**

**Bond**	**ρ**	**$\nabla^2\rho$**	**R_{ij}**	**d_1**	**d_2**	**λ_1**	**λ_2**	**λ_3**	**ε**
O1–C3	2.10	–13.0	1.3710	0.7916	0.5794	–18.1	–15.9	21.0	0.14
CRYSTAL09	*1.99*	*–13.2*	*1.3713*	*0.7942*	*0.5771*	*–16.2*	*–14.8*	*17.8*	*0.10*
O1–H1	2.38	–31.6	0.9921	0.7448	0.2472	–35.7	–34.6	38.8	0.03
CRYSTAL09	*2.25*	*–33.3*	*0.9920*	*0.7523*	*0.2397*	*–34.9*	*–34.0*	*35.7*	*0.02*
O2–C5	2.12	–13.1	1.3683	0.7894	0.5789	–18.2	–16.1	21.3	0.13
CRYSTAL09	*2.06*	*–14.3*	*1.3683*	*0.7856*	*0.5826*	*–16.7*	*–15.9*	*18.3*	*0.05*
O2–H2	2.52	–28.4	0.9921	0.7307	0.2614	–36.0	–34.8	42.3	0.03

CRYSTAL09	*2.30*	*–36.7*	*0.9920*	*0.7509*	*0.2411*	*–36.3*	*–35.4*	*35.1*	*0.02*
N3–C19	2.37	–21.2	1.3396	0.7789	0.5607	–18.5	–16.5	13.8	0.13
CRYSTAL09	*2.32*	*–18.8*	*1.3393*	*0.7430*	*0.5963*	*–18.4*	*–16.6*	*16.3*	*0.11*
N3–C20	2.33	–19.9	1.3310	0.7751	0.5560	–19.4	–16.2	15.7	0.20
CRYSTAL09	*2.38*	*–21.0*	*1.3309*	*0.7433*	*0.5877*	*–19.3*	*–17.5*	*15.8*	*0.11*
N1–C8	2.43	–20.4	1.3406	0.7584	0.5823	–21.1	–17.3	18.0	0.22
CRYSTAL09	*2.35*	*–20.7*	*1.3392*	*0.7473*	*0.5919*	*–19.5*	*–16.9*	*15.6*	*0.15*
N1–C12	2.40	–18.8	1.3435	0.7509	0.5926	–20.8	–16.8	18.7	0.24
CRYSTAL09	*2.37*	*–20.3*	*1.3426*	*0.7383*	*0.6044*	*–19.6*	*–17.2*	*16.6*	*0.14*
N2–C15	2.43	–20.3	1.3410	0.7566	0.5844	–21.1	–17.3	18.1	0.22
CRYSTAL09	*2.33*	*–19.3*	*1.3397*	*0.7373*	*0.6024*	*–19.2*	*–16.6*	*16.5*	*0.15*
N2–C16	2.40	–18.8	1.3420	0.7495	0.5926	–20.7	–16.8	18.7	0.24
CRYSTAL09	*2.33*	*–19.6*	*1.3409*	*0.7430*	*0.5979*	*–19.0*	*–16.7*	*16.1*	*0.14*
C1–C2	2.11	–18.1	1.3937	0.6751	0.7187	–16.0	–13.8	11.7	0.16
CRYSTAL09	*2.08*	*–16.8*	*1.3933*	*0.7061*	*0.6872*	*–16.5*	*–12.7*	*12.4*	*0.30*
C1–C6	2.10	–17.6	1.3922	0.6944	0.6978	–16.6	–13.0	12.0	0.28
CRYSTAL09	*2.14*	*–17.9*	*1.3923*	*0.7018*	*0.6905*	*–16.9*	*–13.7*	*12.7*	*0.24*
C1–C7	1.80	–11.0	1.5128	0.7721	0.7407	–12.0	–11.3	12.3	0.07
CRYSTAL09	*1.67*	*–10.1*	*1.5111*	*0.7678*	*0.7433*	*–11.4*	*–11.1*	*12.4*	*0.03*
C2–C3	2.14	–17.8	1.3920	0.6785	0.7135	–18.0	–13.7	13.9	0.31
CRYSTAL09	*2.14*	*–18.8*	*1.3921*	*0.6737*	*0.7185*	*–17.5*	*–13.5*	*12.2*	*0.30*
C2–H2A	1.99	–21.1	1.0830	0.7167	0.3663	–20.1	–18.9	17.9	0.06
CRYSTAL09	*1.94*	*–21.7*	*1.0830*	*0.6899*	*0.3931*	*–19.1*	*–17.9*	*15.3*	*0.07*
C3–C4	2.19	–19.3	1.3964	0.7297	0.6667	–18.2	–13.9	12.8	0.32
CRYSTAL09	*2.13*	*–18.0*	*1.3963*	*0.7299*	*0.6664*	*–17.2*	*–13.5*	*12.6*	*0.28*
C4–C5	2.22	–19.5	1.4007	0.6705	0.7302	–18.3	–14.3	13.0	0.28
CRYSTAL09	*2.11*	*–17.2*	*1.4007*	*0.6893*	*0.7113*	*–17.1*	*–13.1*	*12.9*	*0.31*
C4–H4	1.96	–21.8	1.0830	0.7275	0.3555	–20.3	–19.1	17.5	0.06
CRYSTAL09	*1.86*	*–17.4*	*1.0830*	*0.7046*	*0.3784*	*–18.0*	*–16.9*	*17.5*	*0.06*
C5–C6	2.15	–18.2	1.3932	0.7192	0.6739	–17.9	–14.1	13.8	0.28
CRYSTAL09	*2.14*	*–18.6*	*1.3932*	*0.7146*	*0.6786*	*–17.4*	*–13.7*	*12.5*	*0.27*
C6–H6	2.02	–20.7	1.0830	0.6984	0.3847	–19.9	–18.7	17.9	0.07
CRYSTAL09	*1.93*	*–20.6*	*1.0830*	*0.6801*	*0.4029*	*–18.9*	*–17.0*	*15.3*	*0.11*
C7–H7A	1.85	–18.0	1.0776	0.6591	0.4185	–16.6	–15.7	14.4	0.06
CRYSTAL09	*1.90*	*–19.8*	*1.0770*	*0.6704*	*0.4066*	*–17.3*	*–16.8*	*14.3*	*0.02*
C7–H7B	1.78	–14.4	1.0771	0.7042	0.3729	–16.3	–15.3	17.2	0.07
CRYSTAL09	*1.92*	*–20.2*	*1.0771*	*0.6813*	*0.3957*	*–17.8*	*–17.6*	*15.2*	*0.01*
C7–H7C	1.78	–13.4	1.0774	0.6908	0.3865	–15.7	–14.9	17.2	0.06
CRYSTAL09	*1.92*	*–20.2*	*1.0771*	*0.6875*	*0.3896*	*–18.2*	*–17.8*	*15.7*	*0.02*
C8–C9	2.21	–18.9	1.3878	0.7210	0.6668	–18.4	–14.1	13.7	0.30
CRYSTAL09	*2.21*	*–20.6*	*1.3870*	*0.7122*	*0.6748*	*–17.7*	*–14.4*	*11.5*	*0.24*
C8–H8	1.81	–15.7	1.0833	0.7078	0.3754	–17.9	–16.2	18.5	0.10

CRYSTAL09	*2.00*	*–23.1*	*1.0831*	*0.7144*	*0.3687*	*–20.7*	*–19.3*	*16.9*	*0.07*
C9–C10	2.14	–16.9	1.3979	0.6923	0.7057	–17.1	–14.1	14.3	0.21
CRYSTAL09	*2.14*	*–18.7*	*1.3979*	*0.6910*	*0.7069*	*–16.9*	*–13.7*	*11.9*	*0.23*
C9–H9	1.85	–16.3	1.0834	0.7213	0.3622	–18.0	–17.4	19.2	0.03
CRYSTAL09	*1.95*	*–21.4*	*1.0832*	*0.6943*	*0.3889*	*–19.1*	*–18.0*	*15.6*	*0.06*
C10–C11	2.12	–16.9	1.3923	0.6920	0.7003	–16.8	–14.2	14.0	0.18
CRYSTAL09	*2.20*	*–20.4*	*1.3923*	*0.7058*	*0.6865*	*–17.5*	*–14.5*	*11.7*	*0.21*
C10–C13	1.78	–10.9	1.4872	0.7458	0.7414	–13.6	–11.8	14.6	0.15
CRYSTAL09	*1.81*	*–13.0*	*1.4872*	*0.7421*	*0.7451*	*–13.2*	*–11.8*	*12.1*	*0.12*
C11–C12	2.24	–19.3	1.3821	0.6635	0.7186	–18.6	–14.3	13.7	0.30
CRYSTAL09	*2.26*	*–21.8*	*1.3812*	*0.6699*	*0.7113*	*–18.4*	*–14.7*	*11.4*	*0.25*
C11–H11	1.85	–16.3	1.0834	0.7230	0.3604	–18.1	–17.5	19.2	0.03
CRYSTAL09	*1.99*	*–22.8*	*1.0830*	*0.7044*	*0.3786*	*–20.1*	*–18.8*	*16.1*	*0.07*
C12–H12	1.83	–15.7	1.0833	0.7069	0.3764	–18.0	–16.4	18.7	0.10
CRYSTAL09	*2.01*	*–23.6*	*1.0830*	*0.7044*	*0.3786*	*–20.6*	*–19.2*	*16.2*	*0.08*
C13–C14	2.12	–17.2	1.3894	0.6893	0.7001	–16.8	–14.2	13.8	0.19
CRYSTAL09	*2.18*	*–19.1*	*1.3894*	*0.7020*	*0.6874*	*–17.1*	*–13.9*	*11.9*	*0.22*
C13–C17	2.14	–17.0	1.3969	0.6999	0.6970	–17.1	–14.2	14.3	0.21
CRYSTAL09	*2.17*	*–19.0*	*1.3970*	*0.7040*	*0.6930*	*–16.9*	*–14.1*	*12.0*	*0.20*
C14–C15	2.23	–19.1	1.3846	0.6640	0.7206	–18.5	–14.2	13.7	0.30
CRYSTAL09	*2.20*	*–20.4*	*1.3838*	*0.6719*	*0.7119*	*–17.5*	*–14.3*	*11.4*	*0.22*
C14–H14	1.85	–16.3	1.0834	0.7204	0.3631	–18.0	–17.4	19.2	0.03
CRYSTAL09	*1.92*	*–19.9*	*1.0831*	*0.6968*	*0.3862*	*–18.7*	*–17.7*	*16.5*	*0.05*
C15–H15	1.81	–15.7	1.0833	0.7095	0.3738	–18.0	–16.3	18.6	0.10
CRYSTAL09	*1.96*	*–21.1*	*1.0830*	*0.7077*	*0.3753*	*–19.8*	*–18.4*	*17.1*	*0.08*
C16–C17	2.24	–19.2	1.3843	0.7179	0.6664	–18.6	–14.3	13.7	0.30
CRYSTAL09	*2.21*	*–20.5*	*1.3835*	*0.7127*	*0.6708*	*–17.7*	*–14.3*	*11.4*	*0.23*
C16–H16	1.81	–15.7	1.0833	0.7108	0.3725	–18.0	–16.3	18.6	0.10
CRYSTAL09	*1.99*	*–23.7*	*1.0831*	*0.6887*	*0.3944*	*–20.1*	*–18.6*	*15.0*	*0.08*
C17–H17	1.83	–16.4	1.0834	0.7293	0.3541	–18.0	–17.5	19.1	0.03
CRYSTAL09	*1.96*	*–22.0*	*1.0831*	*0.6968*	*0.3863*	*–19.4*	*–18.4*	*15.8*	*0.05*
C18–C19	2.36	–22.2	1.3858	0.6951	0.6907	–17.5	–15.4	10.7	0.14
CRYSTAL09	*2.20*	*–20.1*	*1.3842*	*0.6792*	*0.7050*	*–17.6*	*–14.1*	*11.6*	*0.25*
C18–C22	2.07	–16.8	1.3915	0.6765	0.7150	–16.1	–13.3	12.5	0.21
CRYSTAL09	*2.18*	*–19.7*	*1.3911*	*0.6945*	*0.6966*	*–17.4*	*–14.1*	*11.8*	*0.24*
C18–H18	1.91	–20.1	1.0832	0.7288	0.3544	–18.9	–18.2	17.0	0.04
CRYSTAL09	*1.92*	*–20.3*	*1.0832*	*0.6991*	*0.3841*	*–18.8*	*–17.7*	*16.2*	*0.06*
C19–H19	1.92	–19.9	1.0830	0.6567	0.4263	–17.4	–16.6	14.1	0.05
CRYSTAL09	*1.93*	*–19.6*	*1.0830*	*0.7086*	*0.3744*	*–19.2*	*–17.9*	*17.5*	*0.07*
C20–C21	2.20	–18.9	1.3830	0.6776	0.7054	–16.9	–14.1	12.0	0.20
CRYSTAL09	*2.20*	*–19.7*	*1.3827*	*0.7118*	*0.6709*	*–17.1*	*–14.1*	*11.5*	*0.22*
C20–H20	1.78	–14.2	1.0831	0.7184	0.3647	–17.2	–16.1	19.1	0.07

CRYSTAL09	*1.99*	*–23.4*	*1.0830*	*0.6987*	*0.3843*	*–20.2*	*–18.9*	*15.7*	*0.07*
C21–C22	2.09	–16.7	1.3972	0.6718	0.7254	–16.3	–13.2	12.8	0.23
CRYSTAL09	*2.17*	*–19.0*	*1.3970*	*0.6838*	*0.7132*	*–17.0*	*–14.0*	*12.0*	*0.21*
C21–H21	1.88	–20.4	1.0833	0.7378	0.3455	–18.9	–18.3	16.8	0.04
CRYSTAL09	*1.93*	*–19.4*	*1.0830*	*0.7007*	*0.3823*	*–18.7*	*–17.8*	*17.1*	*0.05*

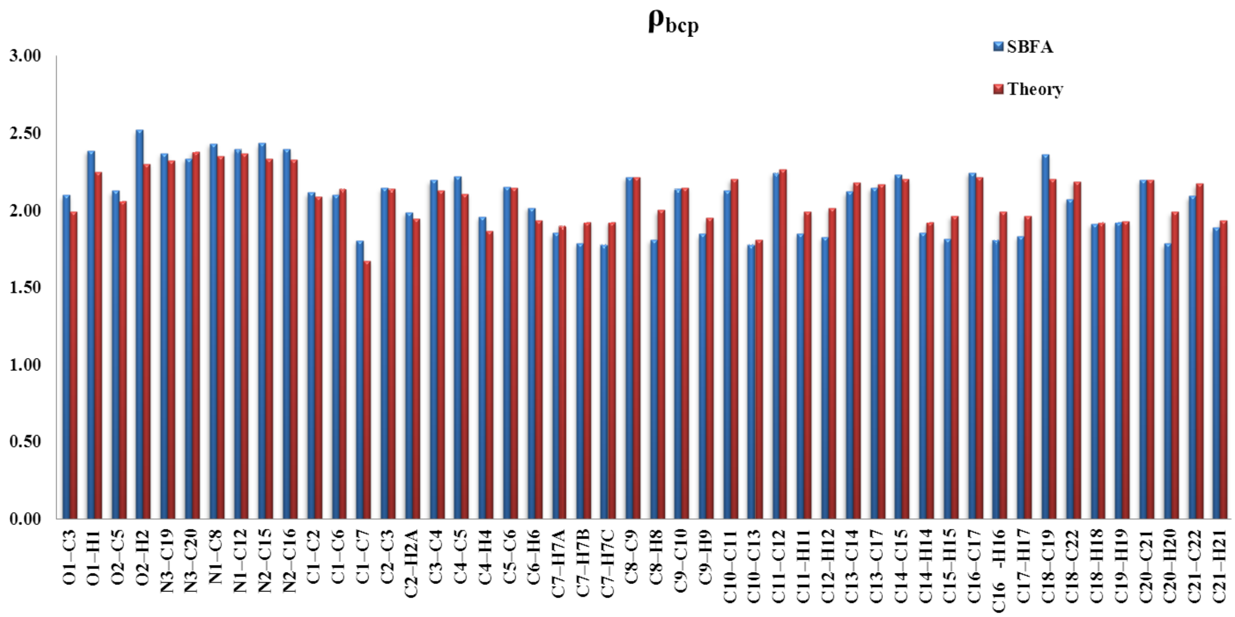

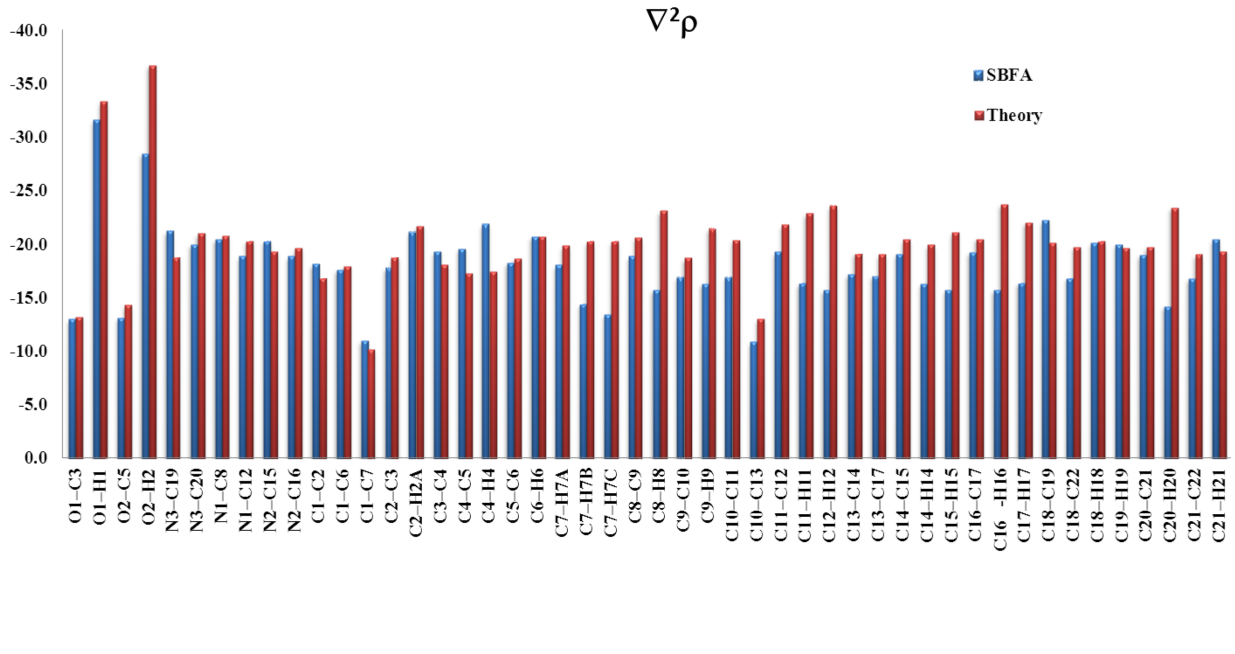

Table 9: Numerical (top) and graphical (bottom) comparison of the *bcp* properties for intramolecular bonding region obtained from SBFA modeled data with that obtained from theoretical calculations performed at B3LYP/6-31G(d,p) level of theory (CRYSTAL09) for Form **III**

**Bond**	**ρ**	$\nabla^2\rho$	R_{ij}	d_1	d_2	λ_1	λ_2	λ_3	**ε**
O1–C3	2.13	–13.3	1.3670	0.7902	0.5768	–18.4	–16.2	21.2	0.13
CRYSTAL09	2.06	–14.2	1.3675	0.7847	0.5828	–16.4	–15.0	17.1	0.10
O1–H1	2.35	–32.7	0.9921	0.7495	0.2425	–35.9	–34.8	37.9	0.03
CRYSTAL09	2.21	–31.1	0.9920	0.7514	0.2406	–33.6	–32.9	35.3	0.02
O2–C5	2.16	–13.2	1.3655	0.7871	0.5783	–18.5	–16.3	21.6	0.13
CRYSTAL09	2.05	–16.0	1.3656	0.7986	0.5671	–16.4	–15.1	15.6	0.09
O2–H2	2.35	–32.8	0.9921	0.7501	0.2420	–35.9	–34.8	37.8	0.03
CRYSTAL09	2.28	–34.8	0.9921	0.7500	0.2421	–35.1	–34.4	34.7	0.02
N1–C15	2.38	–19.5	1.3269	0.7658	0.5611	–19.5	–16.5	16.6	0.18
CRYSTAL09	2.42	–23.3	1.3268	0.7544	0.5724	–19.4	–18.1	14.3	0.07
N1–C16	2.41	–19.4	1.3375	0.7490	0.5885	–20.9	–16.9	18.5	0.24
CRYSTAL09	2.36	–21.3	1.3365	0.7630	0.5735	–19.1	–17.1	14.8	0.12
N2–C8	2.42	–19.5	1.3346	0.7619	0.5727	–19.4	–16.4	16.3	0.18
CRYSTAL09	2.30	–21.4	1.3338	0.7796	0.5542	–18.7	–16.2	13.5	0.15
N2–C12	2.41	–19.8	1.3348	0.7494	0.5853	–21.0	–16.9	18.1	0.24
CRYSTAL09	2.44	–24.0	1.3339	0.7602	0.5736	–20.7	–17.8	14.4	0.17
N3–C19	2.48	–22.2	1.3212	0.7502	0.5709	–21.9	–17.3	17.1	0.26
CRYSTAL09	2.42	–23.1	1.3209	0.7550	0.5659	–20.0	–17.2	14.2	0.16
N3–C20	2.46	–20.4	1.3204	0.7584	0.5620	–20.2	–17.0	16.8	0.18
CRYSTAL09	2.46	–23.3	1.3208	0.7535	0.5673	–19.7	–18.1	14.5	0.09
C1–C2	2.14	–18.1	1.3862	0.6771	0.7090	–16.3	–13.7	11.9	0.19
CRYSTAL09	2.15	–18.6	1.3857	0.7238	0.6619	–16.2	–13.2	10.9	0.23
C1–C6	2.18	–18.6	1.3795	0.6865	0.6929	–17.2	–13.5	12.1	0.27
CRYSTAL09	2.18	–19.3	1.3796	0.6877	0.6919	–16.7	–13.4	10.7	0.25
C1–C7	1.84	–10.4	1.5104	0.7653	0.7451	–12.2	–11.2	13.0	0.09
CRYSTAL09	1.69	–10.7	1.5072	0.7657	0.7415	–11.6	–11.0	11.8	0.05
C2–C3	2.23	–18.9	1.3921	0.6731	0.7190	–18.5	–14.6	14.2	0.27
CRYSTAL09	2.16	–19.3	1.3919	0.6671	0.7248	–16.9	–13.4	11.0	0.26
C2–H2A	1.83	–14.3	1.0830	0.7234	0.3596	–17.7	–16.6	20.0	0.07
CRYSTAL09	1.92	–20.1	1.0831	0.6944	0.3887	–18.4	–17.4	15.7	0.06
C3–C4	2.23	–18.7	1.3882	0.7052	0.6830	–18.4	–14.0	13.7	0.31
CRYSTAL09	2.17	–19.4	1.3881	0.7146	0.6735	–17.3	–13.4	11.3	0.29
C4–C5	2.20	–19.1	1.3899	0.6671	0.7228	–17.8	–14.4	13.0	0.24
CRYSTAL09	2.18	–19.4	1.3888	0.6662	0.7226	–17.4	–13.3	11.3	0.31
C4–H4	2.00	–20.7	1.0830	0.6993	0.3838	–19.5	–18.7	17.5	0.05
CRYSTAL09	1.81	–15.2	1.0830	0.7177	0.3653	–17.1	–16.1	18.1	0.06

C5–C6	2.22	–18.7	1.3949	0.7264	0.6686	–18.4	–14.5	14.2	0.27
CRYSTAL09	2.12	–18.8	1.3937	0.7264	0.6673	–16.6	–12.9	10.7	0.28
C6–H6	1.86	–14.3	1.0830	0.7114	0.3717	–17.8	–16.6	20.1	0.07
CRYSTAL09	1.91	–20.3	1.0832	0.6963	0.3869	–18.5	–17.1	15.4	0.08
C7–H7A	1.84	–21.5	1.0772	0.7316	0.3456	–18.8	–18.1	15.4	0.04
CRYSTAL09	1.84	–15.7	1.0770	0.6961	0.3810	–17.1	–16.2	17.5	0.06
C7–H7B	1.57	–12.1	1.0788	0.7257	0.3531	–14.6	–13.9	16.3	0.05
CRYSTAL09	1.86	–16.8	1.0769	0.6735	0.4034	–16.8	–15.9	15.9	0.06
C7–H7C	1.75	–15.3	1.0772	0.7343	0.3430	–16.7	–16.0	17.4	0.04
CRYSTAL09	1.84	–17.4	1.0772	0.6688	0.4084	–16.9	–15.6	15.1	0.08
C8–C9	2.15	–18.1	1.3776	0.6732	0.7044	–16.8	–13.8	12.5	0.22
CRYSTAL09	2.23	–19.8	1.3777	0.7076	0.6701	–17.2	–14.4	11.8	0.20
C8–H8	1.85	–21.3	1.0830	0.7329	0.3501	–19.2	–18.5	16.4	0.04
CRYSTAL09	1.94	–19.6	1.0827	0.7163	0.3665	–19.3	–18.3	18.0	0.06
C9–C10	2.16	–17.4	1.3834	0.6958	0.6875	–17.3	–14.1	14.1	0.23
CRYSTAL09	2.16	–18.5	1.3828	0.6574	0.7254	–16.1	–13.5	11.2	0.19
C9–H9	1.74	–14.8	1.0830	0.7519	0.3312	–17.5	–16.5	19.3	0.06
CRYSTAL09	1.93	–19.9	1.0832	0.6806	0.4026	–17.9	–17.3	15.3	0.04
C11–C10	2.16	–17.5	1.3888	0.6713	0.7175	–16.8	–13.8	13.2	0.21
CRYSTAL09	2.16	–18.7	1.3887	0.6988	0.6899	–16.6	–13.5	11.4	0.22
C11–C12	2.36	–22.5	1.3775	0.6717	0.7058	–19.2	–15.9	12.6	0.21
CRYSTAL09	2.24	–21.3	1.3759	0.6551	0.7208	–17.9	–14.3	10.9	0.25
C11–H11	1.87	–19.9	1.0832	0.7159	0.3673	–18.7	–17.7	16.5	0.05
CRYSTAL09	1.98	–22.4	1.0831	0.6958	0.3873	–19.1	–18.5	15.3	0.03
C10–C13	1.80	–11.0	1.4837	0.7445	0.7392	–13.7	–11.9	14.7	0.15
CRYSTAL09	1.77	–12.3	1.4836	0.7551	0.7285	–12.5	–11.4	11.6	0.10
C12–H12	1.81	–15.7	1.0833	0.7059	0.3774	–17.9	–16.2	18.5	0.11
CRYSTAL09	1.98	–22.0	1.0831	0.6940	0.3891	–20.0	–18.2	16.2	0.10
C13–C14	2.16	–17.4	1.3892	0.7102	0.6790	–16.8	–13.8	13.3	0.22
CRYSTAL09	2.16	–18.5	1.3890	0.7066	0.6825	–16.4	–13.6	11.5	0.21
C13–C17	2.14	–17.4	1.3847	0.6966	0.6881	–16.5	–13.9	13.1	0.18
CRYSTAL09	2.16	–18.3	1.3850	0.7248	0.6601	–16.4	–13.0	11.2	0.26
C14–C15	2.24	–19.5	1.3803	0.7040	0.6762	–17.6	–14.6	12.7	0.21
CRYSTAL09	2.21	–19.7	1.3797	0.6638	0.7159	–17.1	–14.4	11.7	0.19
C14–H14	1.84	–20.5	1.0832	0.7319	0.3513	–18.8	–17.9	16.3	0.05
CRYSTAL09	1.94	–19.9	1.0831	0.6957	0.3874	–18.6	–18.0	16.6	0.03
C15–H15	1.80	–14.3	1.0831	0.7234	0.3597	–17.4	–16.3	19.5	0.07
CRYSTAL09	1.91	–19.2	1.0829	0.6850	0.3979	–18.4	–16.9	16.1	0.09
C16–C17	2.40	–22.9	1.3758	0.7014	0.6743	–19.5	–16.2	12.8	0.21
CRYSTAL09	2.21	–19.8	1.3747	0.7174	0.6574	–17.3	–14.0	11.4	0.24
C16–H16	1.75	–15.7	1.0833	0.7238	0.3595	–17.7	–16.1	18.1	0.10
CRYSTAL09	1.97	–21.8	1.0831	0.6887	0.3944	–19.6	–18.1	15.8	0.09

C17–H17	1.93	–19.9	1.0832	0.7088	0.3744	–19.0	–18.0	17.1	0.05
CRYSTAL09	1.93	–20.6	1.0831	0.7158	0.3673	–18.7	–18.6	16.8	0.01
C18–C19	2.38	–22.5	1.3808	0.6762	0.7046	–19.2	–16.0	12.8	0.20
CRYSTAL09	2.24	–21.0	1.3789	0.6740	0.7049	–17.6	–14.4	11.0	0.22
C18–C22	2.18	–17.7	1.3839	0.6734	0.7105	–17.0	–14.0	13.2	0.22
CRYSTAL09	2.22	–20.9	1.3831	0.6851	0.6980	–17.0	–14.7	10.8	0.15
C18–H18	1.82	–20.7	1.0832	0.7373	0.3459	–18.8	–17.9	16.1	0.05
CRYSTAL09	1.87	–17.7	1.0834	0.6912	0.3922	–17.6	–16.3	16.2	0.08
C19–H19	1.72	–15.7	1.0833	0.7286	0.3547	–17.5	–16.0	17.8	0.10
CRYSTAL09	1.90	–18.9	1.0831	0.7389	0.3442	–19.7	–18.5	19.3	0.06
C20–C21	2.20	–18.1	1.3797	0.6970	0.6827	–16.9	–14.1	12.9	0.20
CRYSTAL09	2.20	–19.4	1.3795	0.6999	0.6796	–17.2	–13.8	11.6	0.25
C20–H20	1.68	–15.1	1.0831	0.7626	0.3205	–17.3	–16.3	18.5	0.06
CRYSTAL09	1.97	–22.1	1.0830	0.6819	0.4012	–19.3	–17.9	15.1	0.08
C21–C22	2.27	–19.6	1.3748	0.6996	0.6752	–18.0	–14.9	13.3	0.21
CRYSTAL09	2.20	–20.3	1.3754	0.7159	0.6595	–17.2	–14.0	10.9	0.23
C21–H21	1.80	–21.2	1.0832	0.7482	0.3349	–19.1	–18.2	16.0	0.05
CRYSTAL09	1.90	–18.3	1.0831	0.6904	0.3928	–17.8	–16.9	16.5	0.05

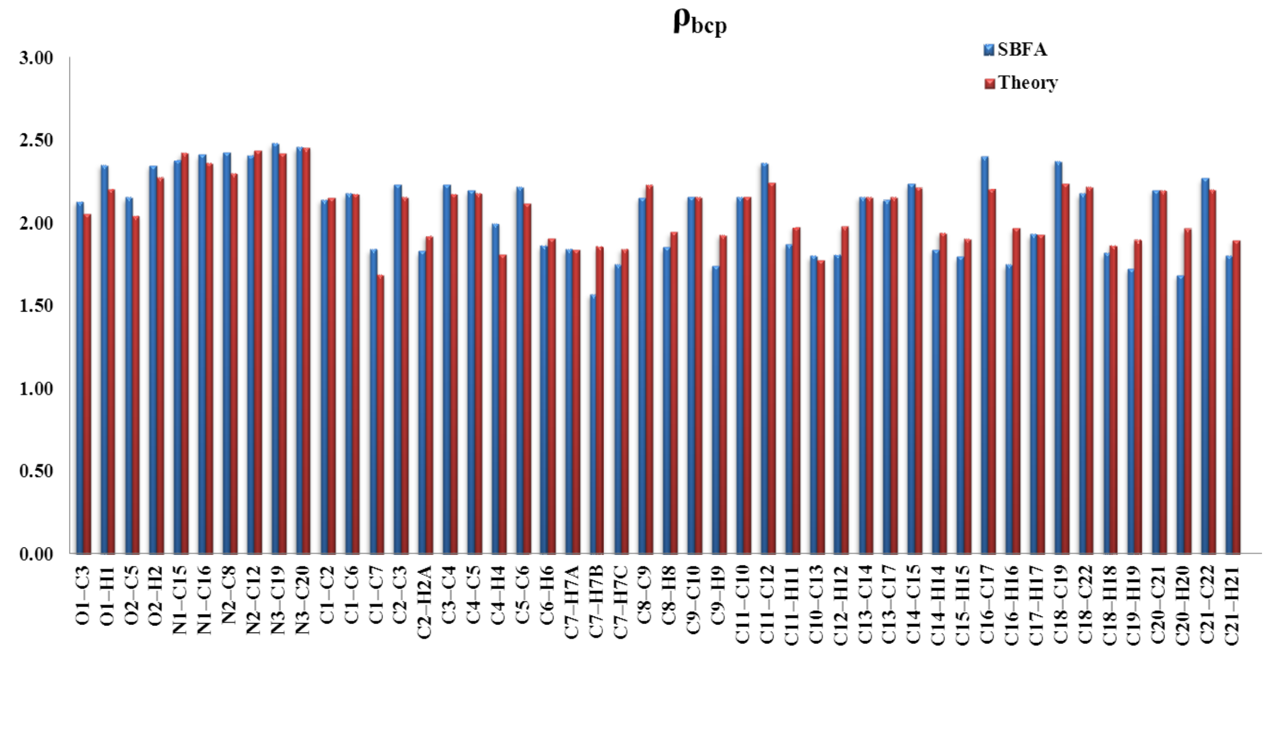

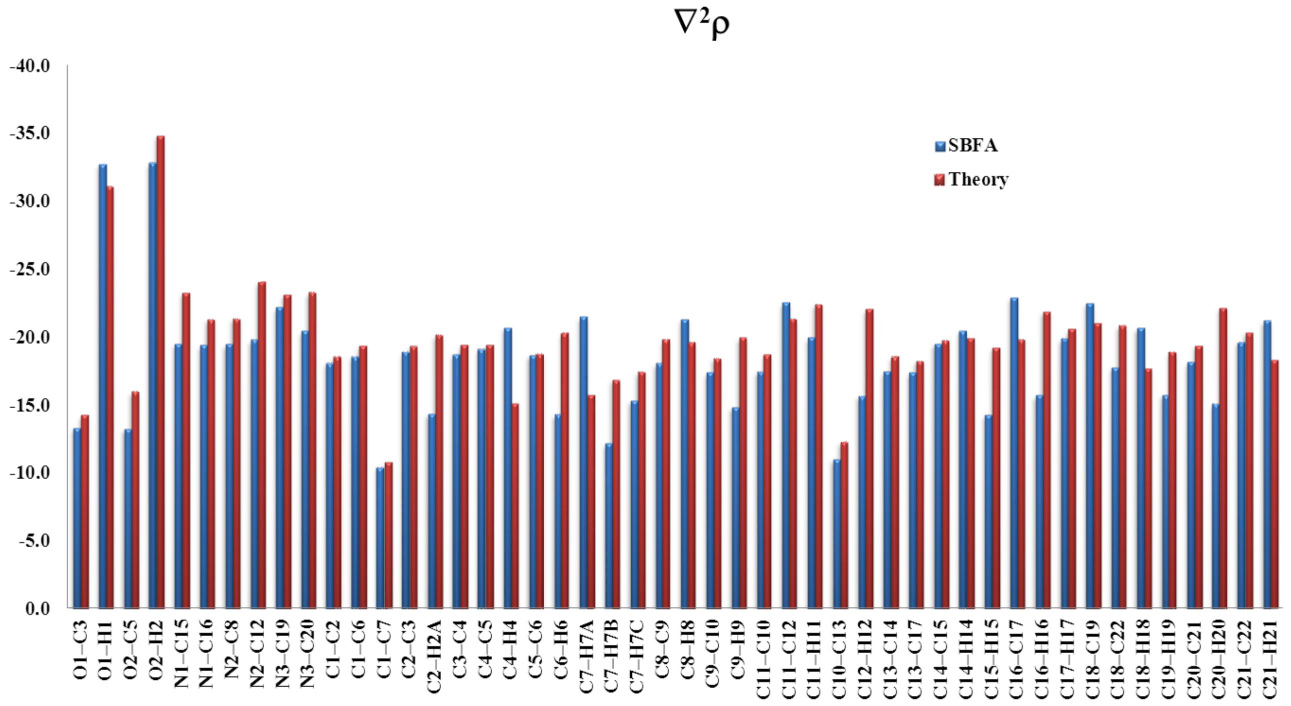

Table 10: Topological values at the *bcp* for intramolecular bonding region obtained from SBFA modeled data for Form **IV**

Bond	ρ	$\nabla^2\rho$	R_{ij}	d_1	d_2	λ_1	λ_2	λ_3	ε
C13–C14	2.18	–18.9	1.3915	0.6733	0.7182	–17.7	–14.2	13.0	0.24
C14 –O1	2.12	–13.3	1.3662	0.5767	0.7895	–18.3	–16.1	21.1	0.13
C15–C14	2.21	–18.7	1.3937	0.6725	0.7212	–18.4	–14.5	14.1	0.27
H1O–O1	2.26	–35.0	0.9921	0.2329	0.7592	–35.7	–34.7	35.4	0.03
C12–O2	2.12	–13.5	1.3641	0.5755	0.7886	–18.4	–16.1	21.0	0.14
C13–C12	2.22	–18.5	1.3898	0.6857	0.7040	–18.3	–13.9	13.7	0.32
C17–C12	2.21	–18.9	1.3921	0.6707	0.7214	–18.4	–14.5	14.0	0.27
H2O–O2	2.34	–32.3	0.9921	0.2437	0.7483	–35.5	–34.4	37.6	0.03
C36–C37	2.17	–18.3	1.3919	0.6792	0.7127	–18.2	–14.2	14.0	0.28
C37–O3	2.12	–13.1	1.3653	0.5790	0.7862	–18.3	–16.1	21.3	0.14
C38–C37	2.21	–19.4	1.3931	0.6606	0.7324	–17.9	–14.5	13.0	0.23
H3O–O3	2.32	–32.7	0.9921	0.2422	0.7499	–35.4	–34.4	37.1	0.03
C34–C39	2.21	–18.7	1.3940	0.6776	0.7164	–18.3	–14.5	14.1	0.27
C38–C39	2.20	–18.5	1.3902	0.6872	0.7030	–18.2	–13.8	13.5	0.32
C39–O4	2.10	–13.7	1.3654	0.5733	0.7921	–18.2	–16.1	20.6	0.13
H4O–O4	2.33	–32.9	0.9921	0.2413	0.7507	–35.7	–34.6	37.3	0.03

C56–C55	2.19	–19.2	1.3957	0.6678	0.7279	–17.8	–14.4	13.0	0.23
C54–C55	2.16	–18.4	1.3902	0.6839	0.7063	–18.2	–14.1	13.9	0.28
C55–O5	2.11	–13.9	1.3643	0.5715	0.7928	–18.3	–16.1	20.5	0.13
H5O–O5	2.32	–33.3	0.9921	0.2398	0.7523	–35.7	–34.7	37.1	0.03
C51–O6	2.11	–13.5	1.3666	0.5757	0.7910	–18.2	–16.1	20.9	0.13
C52–C51	2.21	–18.8	1.3935	0.6755	0.7180	–18.4	–14.5	14.1	0.27
C56–C51	2.21	–18.5	1.3921	0.6872	0.7049	–18.3	–13.9	13.6	0.32
H6O–O6	2.34	–32.6	0.9921	0.2428	0.7492	–35.7	–34.6	37.7	0.03
C9–N1	2.39	–19.0	1.3416	0.5914	0.7502	–20.8	–16.8	18.5	0.24
C10–N1	2.46	–20.2	1.3397	0.5868	0.7529	–21.2	–17.4	18.5	0.22
C1–N2	2.41	–19.4	1.3374	0.5892	0.7482	–21.0	–16.9	18.5	0.24
C3 –N2	2.46	–20.7	1.3359	0.5828	0.7531	–21.4	–17.5	18.2	0.22
C25–N3	2.44	–20.3	1.3390	0.5857	0.7533	–21.2	–17.4	18.3	0.22
C27–N3	2.38	–18.9	1.3428	0.5928	0.7499	–20.7	–16.7	18.5	0.24
C18–N4	2.39	–18.3	1.3441	0.5983	0.7457	–20.7	–16.7	19.0	0.23
C22–N4	2.41	–19.8	1.3435	0.5876	0.7558	–20.9	–17.1	18.2	0.22
C28–N5	2.42	–19.8	1.3354	0.5859	0.7495	–21.0	–17.0	18.3	0.24
C32–N5	2.50	–21.4	1.3307	0.5787	0.7520	–21.6	–17.8	18.1	0.22
H33C–N6	0.04	0.5	2.8326	1.2245	1.6081	–0.1	–0.1	0.8	0.42
C47–N6	2.44	–20.1	1.3413	0.5865	0.7547	–21.1	–17.3	18.4	0.22
C48–N6	2.42	–19.1	1.3399	0.5920	0.7479	–20.9	–16.9	18.7	0.24
C40–N7	2.46	–20.3	1.3382	0.5857	0.7525	–21.3	–17.5	18.4	0.22
C44–N7	2.41	–19.3	1.3382	0.5900	0.7482	–20.9	–16.9	18.5	0.24
C63–N8	2.38	–18.4	1.3446	0.5967	0.7479	–20.6	–16.7	18.8	0.24
C65–N8	2.45	–19.9	1.3393	0.5890	0.7502	–21.2	–17.4	18.6	0.21
C60–N9	2.44	–20.5	1.3386	0.5832	0.7554	–21.2	–17.4	18.1	0.22
C66–N9	2.38	–18.7	1.3438	0.5935	0.7503	–20.7	–16.7	18.6	0.24
C2–C1	2.22	–19.2	1.3848	0.6595	0.7253	–18.4	–14.2	13.5	0.30
C2–C5	2.17	–17.8	1.3862	0.6833	0.7028	–17.4	–14.4	14.0	0.21
H1–C1	1.80	–15.7	1.0833	0.3706	0.7126	–17.9	–16.3	18.5	0.10
H4–C4	1.81	–16.4	1.0834	0.3518	0.7316	–18.0	–17.4	19.0	0.03
C6–C5	1.78	–10.8	1.4882	0.7422	0.7460	–13.6	–11.8	14.6	0.16
H2–C2	1.87	–16.2	1.0835	0.3729	0.7106	–18.0	–17.4	19.2	0.04
H3–C3	1.82	–15.7	1.0833	0.3724	0.7109	–18.1	–16.4	18.7	0.10
C4–C3	2.26	–19.6	1.3797	0.6608	0.7189	–18.7	–14.5	13.6	0.29
C5–C4	2.15	–17.6	1.3849	0.6877	0.6972	–17.1	–14.4	13.9	0.19
C6–C7	2.12	–17.2	1.3899	0.6909	0.6990	–16.8	–14.1	13.8	0.19
C8–C6	2.14	–17.0	1.3968	0.6963	0.7005	–17.1	–14.2	14.3	0.20
H7–C7	1.84	–16.3	1.0834	0.3623	0.7212	–18.0	–17.4	19.1	0.03
H8–C8	1.86	–16.3	1.0834	0.3637	0.7197	–18.1	–17.5	19.4	0.03
C7–C9	2.24	–19.4	1.3800	0.6603	0.7198	–18.6	–14.4	13.5	0.30
H9–C9	1.80	–15.7	1.0833	0.3710	0.7123	–17.9	–16.2	18.5	0.10

H10–C10	1.86	–15.8	1.0833	0.3786	0.7047	–18.3	–16.6	19.1	0.10
C8–C10	2.26	–19.3	1.3808	0.6603	0.7206	–18.6	–14.4	13.8	0.29
C16–C17	2.11	–17.7	1.3911	0.6819	0.7092	–16.0	–13.5	11.9	0.19
C16–C15	2.14	–17.9	1.3904	0.6890	0.7015	–16.8	–13.2	12.2	0.27
C11–C16	1.85	–11.7	1.5043	0.7364	0.7679	–12.7	–11.4	12.3	0.11
H11A–C11	1.80	–14.6	1.0772	0.3742	0.7029	–16.5	–15.3	17.3	0.08
H11B–C11	1.75	–13.6	1.0773	0.3745	0.7029	–15.7	–14.9	17.1	0.06
H11C–C11	1.74	–12.8	1.0776	0.4182	0.6593	–15.0	–14.0	16.1	0.07
H13–C13	1.98	–20.7	1.0830	0.3797	0.7033	–19.5	–18.7	17.5	0.05
H15–C15	1.88	–14.3	1.0831	0.3750	0.7081	–17.9	–16.7	20.2	0.07
H17–C17	1.86	–14.3	1.0831	0.3722	0.7108	–17.8	–16.6	20.1	0.07
C19–C18	2.27	–19.5	1.3805	0.6632	0.7173	–18.8	–14.6	13.8	0.29
C20–C19	2.13	–17.2	1.3899	0.6854	0.7045	–16.9	–14.2	13.9	0.19
H18–C18	1.86	–15.8	1.0833	0.3802	0.7031	–18.3	–16.5	19.0	0.10
H21–C21	1.82	–16.3	1.0834	0.3577	0.7258	–17.9	–17.3	18.9	0.03
C23–C20	1.76	–10.8	1.4883	0.7426	0.7457	–13.6	–11.6	14.4	0.17
H19–C19	1.86	–16.3	1.0834	0.3596	0.7238	–18.2	–17.6	19.5	0.03
H22–C22	1.82	–15.7	1.0833	0.3773	0.7060	–18.0	–16.3	18.5	0.10
C21–C20	2.13	–17.1	1.3955	0.6928	0.7027	–17.1	–14.1	14.1	0.21
C23–C24	2.11	–17.1	1.3905	0.6865	0.7040	–16.8	–14.1	13.8	0.19
C26–C23	2.13	–17.4	1.3914	0.6905	0.7009	–17.1	–14.2	13.9	0.21
C21–C22	2.24	–19.7	1.3781	0.6606	0.7175	–18.7	–14.4	13.4	0.30
H24–C24	1.83	–16.3	1.0834	0.3563	0.7271	–18.0	–17.4	19.1	0.03
H26–C26	1.84	–16.2	1.0834	0.3644	0.7191	–17.9	–17.3	19.0	0.03
C24–C25	2.24	–19.2	1.3829	0.6628	0.7201	–18.6	–14.3	13.7	0.30
H25–C25	1.82	–15.7	1.0833	0.3723	0.7109	–18.0	–16.4	18.7	0.10
H27–C27	1.81	–15.7	1.0833	0.3744	0.7088	–17.9	–16.2	18.5	0.10
C26–C27	2.23	–19.6	1.3790	0.6584	0.7206	–18.6	–14.3	13.4	0.30
C29–C28	2.29	–21.1	1.3839	0.6686	0.7153	–18.6	–15.8	13.3	0.17
H28–C28	1.78	–15.7	1.0833	0.3674	0.7159	–17.8	–16.2	18.4	0.10
H29–C29	1.84	–16.2	1.0835	0.3639	0.7196	–17.9	–17.3	19.0	0.03
H31–C31	1.81	–16.4	1.0834	0.3518	0.7316	–17.9	–17.3	18.9	0.03
C29–C30	2.07	–16.0	1.3821	0.6828	0.6993	–16.9	–12.9	13.9	0.31
H32–C32	1.83	–15.7	1.0833	0.3725	0.7108	–18.2	–16.5	18.9	0.10
C31–C30	2.17	–17.6	1.3899	0.6898	0.7001	–17.4	–14.4	14.2	0.21
C31–C32	2.25	–19.1	1.3824	0.6605	0.7219	–18.6	–14.3	13.8	0.30
C33–C35	1.75	–10.8	1.5083	0.7089	0.7994	–11.7	–10.9	11.8	0.07
C35–C36	2.15	–17.8	1.3911	0.6950	0.6961	–16.9	–13.3	12.4	0.27
C35–C34	2.13	–17.6	1.3930	0.6845	0.7085	–16.2	–13.6	12.1	0.19
H33A–C33	1.80	–13.5	1.0773	0.3852	0.6922	–15.9	–15.1	17.5	0.05
H33B–C33	1.78	–15.0	1.0773	0.3612	0.7161	–16.7	–15.6	17.3	0.07
H33C–C33	1.80	–14.8	1.0771	0.3682	0.7089	–16.5	–15.6	17.3	0.06

H34–C34	1.88	–14.3	1.0830	0.3708	0.7122	–17.9	–16.7	20.3	0.07
H36–C36	1.87	–14.3	1.0831	0.3721	0.7110	–17.8	–16.6	20.1	0.07
H38–C38	1.98	–20.7	1.0830	0.3831	0.6999	–19.4	–18.6	17.3	0.05
C41–C40	2.26	–19.3	1.3810	0.6619	0.7192	–18.7	–14.5	13.8	0.29
C42–C41	2.15	–17.2	1.3941	0.6967	0.6974	–17.2	–14.2	14.2	0.21
H40–C40	1.86	–15.8	1.0833	0.3792	0.7040	–18.3	–16.6	19.1	0.10
H43–C43	1.85	–16.2	1.0834	0.3675	0.7160	–18.0	–17.4	19.1	0.03
C42–C45	1.76	–10.9	1.4865	0.7430	0.7436	–13.5	–11.8	14.4	0.14
H41–C41	1.87	–16.3	1.0834	0.3636	0.7199	–18.2	–17.6	19.5	0.03
H44–C44	1.82	–15.7	1.0833	0.3749	0.7084	–18.0	–16.3	18.7	0.10
C42–C43	2.11	–17.4	1.3864	0.6887	0.6978	–16.8	–14.2	13.6	0.19
C45–C46	2.10	–16.8	1.3932	0.6894	0.7038	–16.7	–14.1	13.9	0.18
C49–C(45	2.14	–17.1	1.3949	0.6945	0.7004	–17.1	–14.2	14.1	0.21
C43–C44	2.22	–19.2	1.3834	0.6598	0.7236	–18.5	–14.2	13.5	0.30
H46–C46	1.89	–16.3	1.0835	0.3705	0.7130	–18.2	–17.6	19.5	0.04
H49–C49	1.87	–16.2	1.0834	0.3684	0.7150	–18.1	–17.5	19.4	0.03
C46–C47	2.25	–19.3	1.3802	0.6601	0.7201	–18.6	–14.4	13.7	0.30
H47–C47	1.87	–15.8	1.0833	0.3841	0.6992	–18.3	–16.6	19.1	0.10
H48–C48	1.85	–15.7	1.0833	0.3785	0.7048	–18.2	–16.5	18.9	0.10
C49–C48	2.25	–19.4	1.3803	0.6593	0.7210	–18.6	–14.4	13.6	0.29
C53–C52	2.13	–17.8	1.3911	0.6847	0.7064	–16.7	–13.2	12.1	0.27
C50–C53	1.83	–11.7	1.5088	0.7385	0.7704	–12.6	–11.2	12.1	0.12
C53–C54	2.10	–17.7	1.3920	0.6776	0.7144	–16.0	–13.5	11.8	0.19
H50A–C50	1.73	–13.1	1.0773	0.4193	0.6579	–14.8	–14.0	15.8	0.06
H50B–C50	1.76	–14.7	1.0771	0.3663	0.7109	–16.2	–15.3	16.8	0.06
H50C–C50	1.74	–13.5	1.0773	0.3776	0.6997	–15.6	–14.7	16.9	0.06
H52–C52	1.88	–14.3	1.0830	0.3725	0.7105	–17.9	–16.7	20.3	0.07
H54–C54	1.82	–14.3	1.0830	0.3602	0.7229	–17.7	–16.5	19.8	0.07
H56–C56	1.97	–20.7	1.0830	0.3780	0.7051	–19.4	–18.6	17.3	0.05
C58–C57	2.12	–17.1	1.3913	0.6906	0.7007	–16.8	–14.2	13.9	0.19
C58–C61	1.79	–10.9	1.4858	0.7415	0.7443	–13.8	–11.8	14.6	0.17
C57–C66	2.25	–19.7	1.3793	0.6620	0.7173	–18.7	–14.5	13.5	0.29
H57–C57	1.83	–16.3	1.0834	0.3574	0.7260	–18.0	–17.4	19.1	0.03
H60–C60	1.84	–15.7	1.0833	0.3787	0.7046	–18.1	–16.4	18.8	0.10
H66–C66	1.76	–15.7	1.0833	0.3625	0.7207	–17.7	–16.1	18.2	0.10
C59–C58	2.15	–17.4	1.3916	0.6904	0.7013	–17.2	–14.3	14.1	0.21
C61–C62	2.12	–17.1	1.3900	0.6939	0.6961	–16.8	–14.2	13.9	0.18
C64–C61	2.17	–17.3	1.3934	0.6930	0.7004	–17.3	–14.3	14.3	0.21
H59–C59	1.84	–16.3	1.0834	0.3618	0.7217	–18.0	–17.4	19.1	0.03
C59–C60	2.25	–19.6	1.3784	0.6592	0.7192	–18.7	–14.4	13.5	0.30
H62–C62	1.84	–16.2	1.0834	0.3625	0.7210	–17.9	–17.3	19.0	0.03
H64–C64	1.83	–16.4	1.0834	0.3531	0.7303	–18.1	–17.5	19.1	0.03

C62–C63	2.23	–19.3	1.3832	0.6599	0.7233	–18.5	–14.3	13.5	0.30
H63–C63	1.81	–15.7	1.0833	0.3708	0.7124	–18.0	–16.3	18.6	0.10
H65–C65	1.87	–15.8	1.0833	0.3818	0.7015	–18.4	–16.6	19.2	0.10
C64–C65	2.28	–19.6	1.3781	0.6597	0.7184	–18.9	–14.6	13.8	0.30

Table 11: Topological values at the *bcp* for intramolecular bonding region obtained from SBFA modeled data for Form **V**

**Bond**	**ρ**	**$\nabla^2\rho$**	**R_{ij}**	**d_1**	**d_2**	**λ_1**	**λ_2**	**λ_3**	**ε**
C54–C55	2.23	–19.1	1.3900	0.6705	0.7195	–18.6	–14.7	14.2	0.26
C55–O1	2.13	–13.1	1.3685	0.5789	0.7895	–18.3	–16.1	21.4	0.13
C56–C55	2.17	–18.0	1.3942	0.6816	0.7126	–18.1	–14.1	14.2	0.29
H1O–O1	2.31	–33.9	0.9921	0.2376	0.7545	–35.9	–34.8	36.8	0.03
C58–C57	2.20	–18.6	1.3974	0.6679	0.7294	–18.2	–14.4	14.1	0.26
C56–C57	2.16	–17.7	1.3930	0.6761	0.7169	–18.1	–13.8	14.2	0.32
C57–O2	2.13	–12.7	1.3707	0.5826	0.7882	–18.3	–16.1	21.7	0.14
H2O–O2	2.43	–30.4	0.9921	0.2518	0.7403	–35.8	–34.7	40.0	0.03
C39–O3	2.08	–13.5	1.3713	0.5739	0.7975	–18.0	–15.9	20.4	0.13
C40–C39	2.18	–18.9	1.3887	0.6896	0.6991	–18.4	–14.4	13.8	0.28
C38–C39	2.20	–18.8	1.3932	0.6811	0.7122	–18.4	–14.4	14.1	0.28
H3O–O3	2.20	–38.0	0.9921	0.2215	0.7706	–36.2	–35.3	33.5	0.03
C38–C43	2.16	–17.9	1.3902	0.6794	0.7108	–18.1	–13.8	14.1	0.31
C42–C43	2.17	–18.0	1.3943	0.6664	0.7280	–18.1	–13.8	14.0	0.31
C43–O4	2.10	–12.8	1.3717	0.5814	0.7903	–18.1	–15.9	21.2	0.14
H4O–O4	2.34	–32.6	0.9921	0.2424	0.7496	–35.7	–34.6	37.6	0.03
C45–C50	2.17	–17.8	1.3925	0.6800	0.7124	–18.2	–13.8	14.3	0.31
C49–C50	2.18	–18.1	1.3939	0.6651	0.7287	–18.2	–13.9	14.1	0.31
C50–O5	2.09	–11.8	1.3786	0.5894	0.7892	–17.9	–15.7	21.8	0.14
H5O–O5	2.33	–32.7	0.9921	0.2419	0.7502	–35.6	–34.6	37.5	0.03
C46–O6	2.10	–12.7	1.3735	0.5816	0.7919	–18.0	–15.9	21.2	0.14
C47–C46	2.21	–18.8	1.3933	0.6721	0.7212	–18.4	–14.5	14.0	0.26
C45–C46	2.18	–18.1	1.3938	0.6873	0.7065	–18.2	–14.2	14.2	0.28
H6O–O6	2.33	–33.2	0.9921	0.2402	0.7518	–35.8	–34.7	37.4	0.03
C12–O7	2.13	–12.3	1.3715	0.5854	0.7861	–18.2	–16.0	21.9	0.14
C13–C12	2.21	–18.8	1.3908	0.6776	0.7132	–18.4	–14.5	14.1	0.27
C11–C12	2.22	–18.7	1.3951	0.6691	0.7260	–18.4	–14.5	14.3	0.27
H7O–O7	2.39	–31.4	0.9921	0.2474	0.7447	–35.7	–34.6	38.8	0.03
C11–C16	2.17	–18.1	1.3886	0.6729	0.7157	–18.2	–13.9	14.0	0.31
C15–C16	2.21	–18.7	1.3961	0.6685	0.7276	–18.3	–14.5	14.1	0.26
C16–O8	2.14	–12.7	1.3712	0.5819	0.7894	–18.3	–16.1	21.7	0.14
H8O–O8	2.39	–32.0	0.9921	0.2454	0.7466	–36.0	–34.9	38.9	0.03

C28–N1	2.43	–20.6	1.3380	0.5819	0.7561	–21.1	–17.3	17.8	0.22
C32–N1	2.39	–18.7	1.3411	0.5944	0.7467	–20.7	–16.8	18.8	0.23
C35–N2	2.40	–19.2	1.3407	0.5901	0.7506	–20.8	–16.8	18.5	0.24
C36–N2	2.46	–20.5	1.3381	0.5845	0.7536	–21.3	–17.5	18.3	0.22
C18–N3	2.46	–20.3	1.3389	0.5855	0.7533	–21.2	–17.5	18.4	0.22
C22–N3	2.38	–18.9	1.3428	0.5906	0.7522	–20.7	–16.7	18.4	0.24
C25–N4	2.45	–20.3	1.3306	0.5826	0.7480	–21.3	–17.2	18.2	0.24
C26–N4	2.53	–23.7	1.3154	0.5606	0.7548	–22.2	–18.2	16.7	0.22
C59–N5	2.40	–17.9	1.3488	0.6000	0.7488	–20.6	–16.7	19.4	0.23
C63–N5	2.46	–20.2	1.3412	0.5856	0.7556	–21.2	–17.4	18.5	0.22
C66–N6	2.42	–19.9	1.3434	0.5877	0.7557	–21.0	–17.2	18.3	0.22
C67–N6	2.40	–18.6	1.3433	0.5955	0.7477	–20.7	–16.8	18.9	0.24
H66–C66	1.81	–15.7	1.0833	0.3716	0.7117	–18.0	–16.3	18.6	0.10
C8 –N7	2.48	–22.5	1.3247	0.5691	0.7556	–21.8	–17.8	17.1	0.22
C10–N7	2.44	–20.2	1.3292	0.5824	0.7469	–21.2	–17.2	18.1	0.23
H9–C9	1.77	–16.7	1.0834	0.3367	0.7468	–17.9	–17.4	18.6	0.03
C1–N8	2.48	–20.8	1.3338	0.5819	0.7519	–21.4	–17.6	18.2	0.21
C5–N8	2.48	–19.9	1.3309	0.5894	0.7415	–21.4	–17.4	18.9	0.23
H2–C2	1.74	–16.9	1.0834	0.3306	0.7528	–17.9	–17.3	18.3	0.03
H5–C5	1.72	–16.1	1.0832	0.3386	0.7446	–17.8	–16.3	18.0	0.09
C2–C1	2.32	–21.0	1.3799	0.6715	0.7084	–18.8	–16.0	13.7	0.18
C2–C3	2.13	–16.8	1.3719	0.6857	0.6862	–17.4	–13.3	13.9	0.30
H1–C1	1.73	–15.9	1.0832	0.3480	0.7352	–17.7	–16.2	18.0	0.09
H4–C4	1.74	–16.9	1.0834	0.3286	0.7548	–17.9	–17.4	18.3	0.03
C6–C3	1.78	–10.9	1.4849	0.7422	0.7427	–13.8	–11.7	14.6	0.18
C3 –C4	2.20	–17.9	1.3840	0.6915	0.6925	–17.6	–14.6	14.3	0.20
C7–C6	2.20	–18.3	1.3780	0.6853	0.6927	–17.6	–14.6	14.0	0.20
C6–C9	2.12	–16.4	1.3753	0.6875	0.6878	–17.2	–13.2	14.0	0.30
C4–C5	2.27	–19.0	1.3850	0.6629	0.7221	–18.7	–14.5	14.1	0.29
H7–C7	1.74	–16.7	1.0834	0.3330	0.7504	–17.7	–17.2	18.2	0.03
C7–C8	2.26	–19.9	1.3770	0.6629	0.7141	–18.7	–14.6	13.5	0.28
H8–C8	1.75	–15.7	1.0833	0.3602	0.7231	–17.6	–16.0	18.0	0.10
H10–C10	1.75	–15.7	1.0833	0.3584	0.7248	–17.7	–16.1	18.0	0.10
C9–C10	2.34	–21.8	1.3761	0.6723	0.7038	–19.0	–16.2	13.4	0.17
H11–C11	1.86	–14.3	1.0830	0.3681	0.7149	–17.8	–16.6	20.1	0.07
C14–C13	2.16	–17.8	1.3900	0.6949	0.6952	–17.0	–13.3	12.4	0.28
H15–C15	1.98	–19.4	1.0831	0.4107	0.6724	–18.7	–17.7	17.0	0.06
H13–C13	2.00	–19.4	1.0831	0.4085	0.6746	–18.9	–17.9	17.4	0.06
C14–C15	2.10	–17.1	1.3975	0.6945	0.7029	–15.9	–13.3	12.1	0.20
C17–C14	1.76	–10.5	1.5168	0.7180	0.7988	–11.8	–10.6	11.9	0.11
H17A–C17	1.88	–18.3	1.0771	0.3886	0.6884	–18.0	–15.9	15.6	0.13
H17B–C17	1.66	–11.6	1.0773	0.3801	0.6972	–15.1	–13.5	17.0	0.12

H17C–C17	1.71	–11.9	1.0774	0.3788	0.6986	–15.6	–13.6	17.3	0.15
C19–C18	2.24	–19.3	1.3831	0.6591	0.7240	–18.6	–14.3	13.6	0.30
C19–C20	2.16	–17.4	1.3903	0.6875	0.7028	–17.3	–14.3	14.2	0.20
H18–C18	1.83	–15.7	1.0833	0.3731	0.7102	–18.1	–16.5	18.9	0.10
H21–C21	1.82	–16.4	1.0834	0.3534	0.7300	–18.0	–17.4	19.0	0.03
C20–C23	1.79	–10.7	1.4901	0.7420	0.7481	–13.8	–11.7	14.8	0.18
H19–C19	1.86	–16.2	1.0834	0.3661	0.7174	–18.0	–17.5	19.3	0.03
H22–C22	1.83	–15.7	1.0833	0.3825	0.7008	–18.0	–16.3	18.6	0.11
C21–C20	2.09	–15.8	1.3850	0.6879	0.6970	–16.9	–13.0	14.1	0.30
C24–C23	2.15	–16.8	1.3746	0.6657	0.7089	–17.7	–13.2	14.1	0.33
C27–C23	2.14	–16.7	1.3723	0.6793	0.6930	–17.4	–13.4	14.1	0.30
C21–C22	2.29	–20.9	1.3852	0.6759	0.7093	–18.6	–15.8	13.5	0.18
H24–C24	1.75	–16.5	1.0834	0.3431	0.7403	–17.6	–17.1	18.2	0.03
H27–C27	1.80	–16.5	1.0834	0.3472	0.7362	–18.0	–17.4	18.9	0.03
C24–C25	2.28	–20.9	1.3856	0.6684	0.7171	–18.5	–15.8	13.4	0.17
H25–C25	1.72	–15.8	1.0832	0.3496	0.7336	–17.6	–16.1	17.8	0.10
H26–C26	1.69	–15.9	1.0832	0.3441	0.7391	–17.4	–15.9	17.5	0.09
C27–C26	2.30	–20.9	1.3836	0.6753	0.7084	–18.6	–15.9	13.6	0.18
C29–C28	2.23	–19.2	1.3818	0.6654	0.7164	–18.5	–14.3	13.6	0.29
C30–C29	2.13	–17.2	1.3939	0.6956	0.6983	–17.1	–14.1	14.1	0.21
H28–C28	1.87	–15.8	1.0833	0.3902	0.6931	–18.2	–16.4	18.8	0.11
H31–C31	1.89	–16.3	1.0835	0.3700	0.7135	–18.2	–17.6	19.6	0.03
C30–C33	1.76	–10.8	1.4891	0.7403	0.7488	–13.6	–11.6	14.4	0.18
H29–C29	1.90	–16.3	1.0835	0.3733	0.7102	–18.3	–17.7	19.7	0.03
H32–C32	1.90	–15.9	1.0833	0.3935	0.6898	–18.4	–16.6	19.1	0.11
C30–C31	2.04	–15.3	1.3888	0.6927	0.6961	–16.6	–12.7	14.0	0.30
C33–C34	2.11	–16.8	1.3957	0.6948	0.7009	–16.7	–14.0	13.9	0.19
C37–C33	2.15	–17.1	1.3948	0.6941	0.7007	–17.1	–14.3	14.3	0.20
C31–C32	2.28	–20.5	1.3882	0.6751	0.7131	–18.4	–15.7	13.7	0.17
H34–C34	1.92	–16.3	1.0835	0.3793	0.7042	–18.3	–17.7	19.7	0.04
H37–C37	1.85	–16.3	1.0834	0.3606	0.7228	–18.1	–17.5	19.3	0.03
C34–C35	2.23	–19.2	1.3839	0.6633	0.7206	–18.5	–14.3	13.6	0.29
H35–C35	1.85	–15.8	1.0833	0.3839	0.6994	–18.2	–16.4	18.8	0.11
H36–C36	1.88	–15.8	1.0833	0.3836	0.6996	–18.3	–16.6	19.2	0.10
C37–C36	2.23	–18.6	1.3897	0.6659	0.7238	–18.4	–14.2	13.9	0.29
H38–C38	1.90	–14.4	1.0831	0.3745	0.7086	–18.1	–16.9	20.6	0.07
C41–C40	2.17	–17.9	1.3898	0.6932	0.6966	–17.0	–13.3	12.4	0.28
H42–C42	1.93	–19.4	1.0831	0.3969	0.6862	–18.6	–17.6	16.8	0.06
H40–C40	1.97	–19.5	1.0831	0.3973	0.6858	–18.9	–17.9	17.3	0.06
C41–C42	2.15	–18.1	1.3919	0.6857	0.7063	–16.2	–14.0	12.1	0.16
C44–C41	1.76	–10.4	1.5182	0.7177	0.8005	–11.7	–10.7	11.9	0.09
H44A–C44	1.87	–17.9	1.0772	0.3881	0.6891	–17.8	–15.8	15.8	0.13

H44B–C44	1.70	–12.2	1.0771	0.3759	0.7012	–15.7	–13.7	17.2	0.15
H44C–C44	1.68	–11.7	1.0773	0.3839	0.6933	–15.1	–13.5	16.9	0.12
H45–C45	1.90	–14.4	1.0831	0.3722	0.7109	–18.1	–16.9	20.7	0.07
C47–C48	2.15	–17.7	1.3915	0.6934	0.6982	–16.8	–13.2	12.3	0.27
H49–C49	1.93	–19.5	1.0831	0.3945	0.6886	–18.7	–17.7	16.9	0.06
H47–C47	1.97	–19.4	1.0831	0.4061	0.6770	–18.8	–17.7	17.1	0.06
C48–C49	2.14	–17.9	1.3953	0.6845	0.7108	–16.1	–13.9	12.1	0.16
C51–C48	1.75	–10.4	1.5120	0.7136	0.7985	–11.2	–11.1	11.9	0.01
H51A–C51	1.83	–17.6	1.0772	0.3953	0.6819	–17.3	–15.5	15.2	0.11
H51B–C51	1.70	–12.2	1.0771	0.3769	0.7002	–15.6	–13.6	17.1	0.15
H51C–C51	1.70	–11.8	1.0776	0.3803	0.6973	–15.6	–13.4	17.2	0.16
C52–C53	1.76	–10.8	1.5076	0.7128	0.7948	–11.4	–11.2	11.8	0.02
C53–C54	2.15	–17.6	1.3930	0.6945	0.6986	–16.8	–13.2	12.4	0.27
C53–C58	2.08	–16.9	1.4004	0.6950	0.7054	–15.8	–13.2	12.1	0.20
H52A–C52	1.87	–18.2	1.0771	0.3898	0.6872	–17.9	–15.8	15.5	0.13
H52B–C52	1.68	–11.5	1.0779	0.3768	0.7011	–15.5	–13.3	17.3	0.16
H52C–C52	1.67	–11.7	1.0773	0.3854	0.6919	–15.1	–13.4	16.8	0.12
H54–C54	1.96	–19.4	1.0831	0.3995	0.6836	–18.8	–17.8	17.2	0.06
H56–C56	1.87	–14.3	1.0831	0.3683	0.7147	–17.9	–16.7	20.2	0.07
H58–C58	1.92	–19.4	1.0831	0.3962	0.6869	–18.5	–17.5	16.6	0.06
C60–C59	2.25	–19.4	1.3816	0.6570	0.7246	–18.7	–14.4	13.7	0.30
C61–C60	2.13	–17.1	1.3896	0.6931	0.6965	–16.8	–14.2	13.9	0.18
H59–C59	1.80	–15.8	1.0833	0.3622	0.7210	–18.0	–16.4	18.7	0.10
H60–C60	1.82	–16.3	1.0834	0.3570	0.7264	–17.9	–17.3	19.0	0.03
H61–C62	1.86	–16.3	1.0834	0.3610	0.7224	–18.2	–17.6	19.5	0.03
C61–C64	1.79	–10.7	1.4908	0.7432	0.7476	–13.6	–11.8	14.8	0.15
H63–C63	1.83	–15.7	1.0833	0.3716	0.7117	–18.1	–16.4	18.8	0.10
C62–C61	2.17	–17.3	1.3920	0.6923	0.6997	–17.3	–14.4	14.4	0.20
C65–C64	2.15	–16.9	1.3961	0.6880	0.7081	–17.1	–14.2	14.3	0.20
C64–C68	2.13	–16.7	1.3963	0.6970	0.6993	–16.8	–14.1	14.2	0.19
C62–C63	2.26	–19.2	1.3830	0.6635	0.7195	–18.7	–14.4	13.9	0.29
H65–C65	1.80	–16.3	1.0834	0.3527	0.7308	–17.8	–17.3	18.7	0.03
H68–C68	1.84	–16.3	1.0834	0.3589	0.7245	–18.0	–17.5	19.2	0.03
C65–C66	2.22	–18.9	1.3846	0.6618	0.7228	–18.4	–14.2	13.7	0.30
H67–C67	1.88	–15.8	1.0833	0.3860	0.6973	–18.3	–16.6	19.1	0.11
C68–C67	2.24	–19.1	1.3836	0.6631	0.7206	–18.6	–14.3	13.8	0.30

Scheme 1: Supramolecular synthons and the corresponding in-house SBFA library entry.

Synthon	Charge density SBFA library

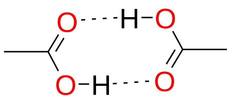<br>**Carboxylic acid O–H···O dimer synthon**	(a) 4-Methoxybenzoic acid (Hathwar *et al.*, 2011)<br>(b) 4-Methylbenzoic acid (Hathwar *et al.*, 2011)<br>(c) 4-(Acetylamino)benzoic acid (Hathwar *et al.*, 2011)<br>(d) 4-Fluorobenzoic acid (Hathwar *et al.*, 2011)
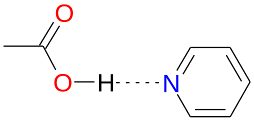<br>**Carboxylic acid–pyridine O–H···N synthon**	Salicylic acid-nicotinamide cocrystal (Hathwar *et al.*, 2010)
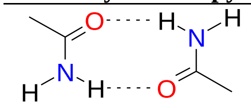<br>**Amide dimer N–H···O synthon**	4-Fluorobenzamide (Hathwar & Guru Row, 2011)
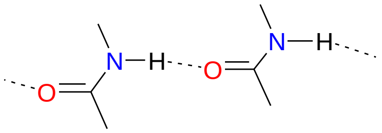<br>**Amide infinite chain**	(a) 4-(Acetylamino)benzoic acid (Hathwar *et al.*, 2011)<br>(b) 4-Methylacetanilide (Hathwar *et al.*, 2011)
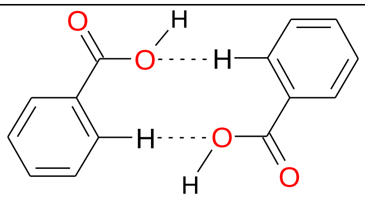<br>**C–H···O dimer synthon**	(a) 4-(Acetylamino)benzoic acid (Hathwar *et al.*, 2011)<br>(b) 4-Fluorobenzoic acid (Hathwar *et al.*, 2011)
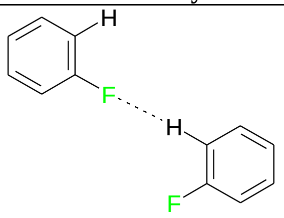<br>**C–H···F infinite chain**	4-Fluorobenzoic acid (Hathwar *et al.*, 2011)
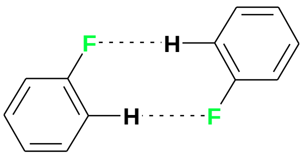<br>**C–H···F dimer**	2-Chloro-4-fluoro-benzoic acid (Hathwar & Guru Row, 2011)
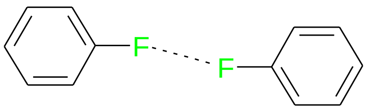<br>**F···F interaction**	4-Fluorobenzamide (Hathwar & Guru Row, 2011)
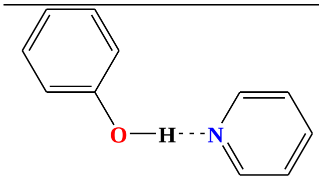<br>**Phenol–pyridine O–H···N synthon**	4-hydroxybenzoic acid–isonicotinamide cocrystal (present study)

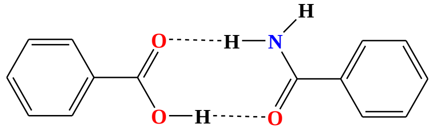 **Carboxylic acid–amide hetero dimer synthon**	4-hydroxybenzoic acid–isonicotinamide cocrystal (present study)
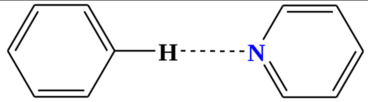 **C–H···N interaction**	(2-chloroquinolin–3–yl)methanol (Hathwar & Guru Row, 2010)

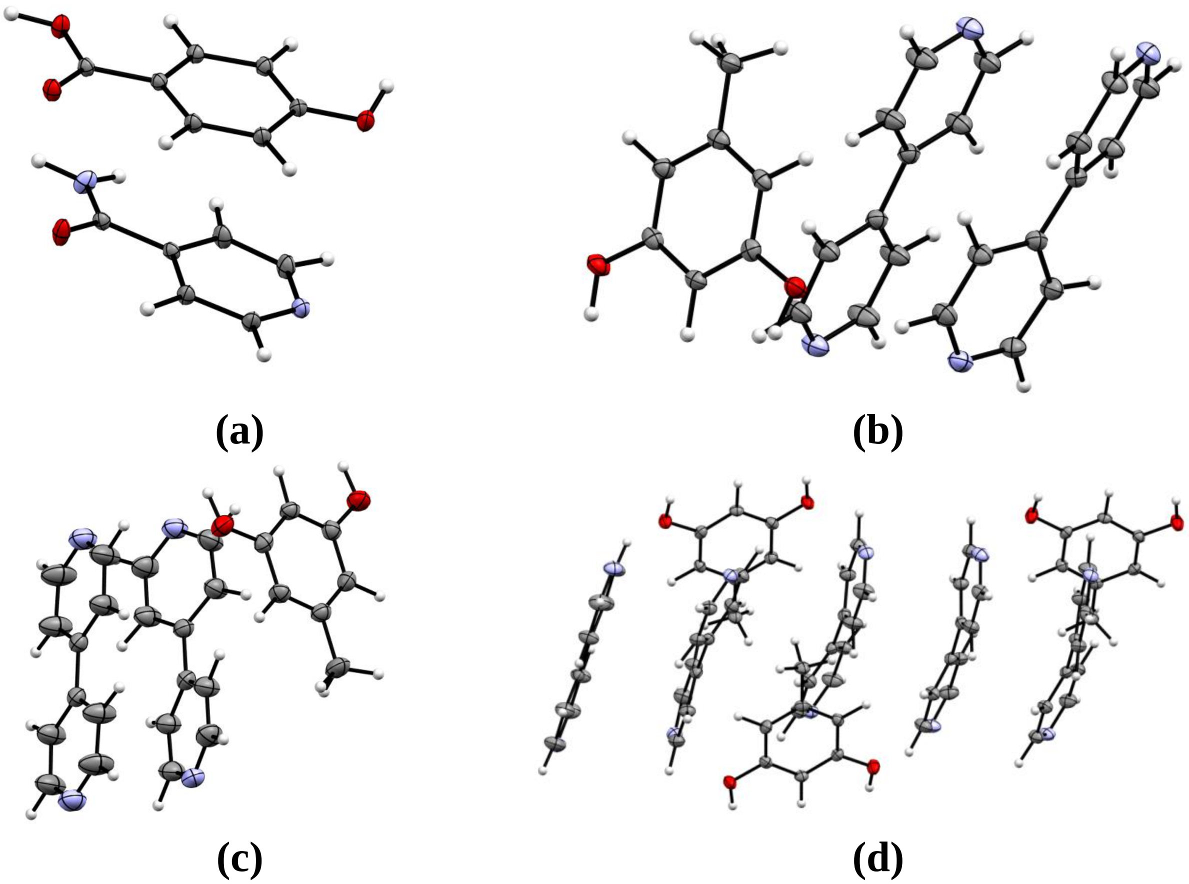

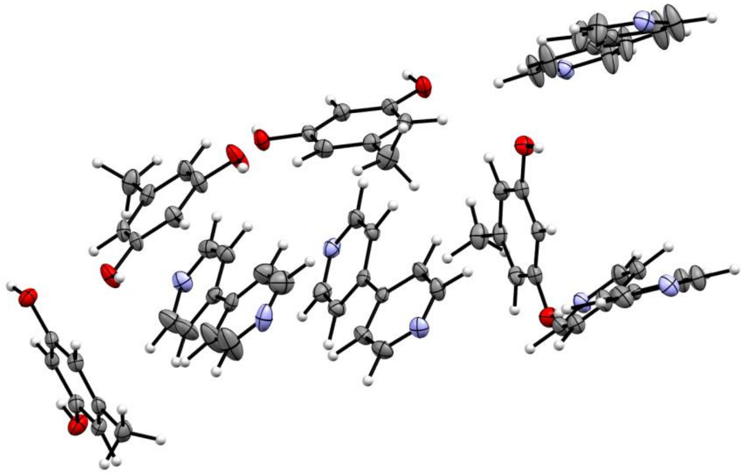

(e)

Fig. 1: ORTEP diagrams for the asymmetric unit of the crystal structures (ellipsoids are drawn at 50% probability) (a) 4-Hydroxybenzoic acid:Isonicotinamide (b) Form **II** (c) Form **III** (d) Form **IV** (e) Form **V**

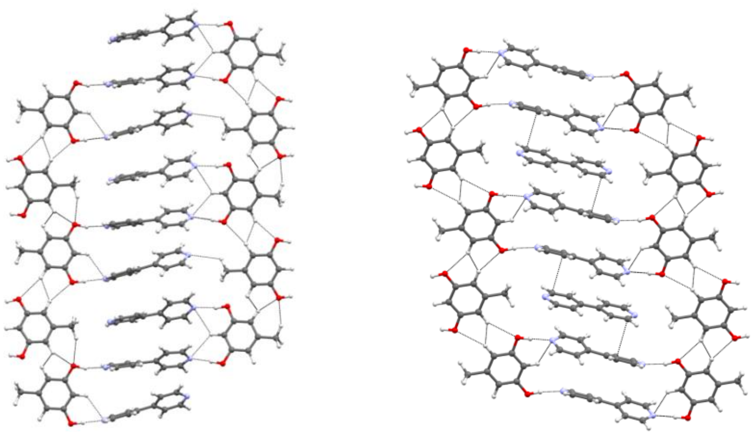

(a) (b)

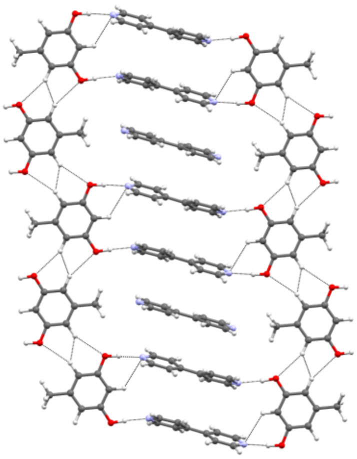

(c)

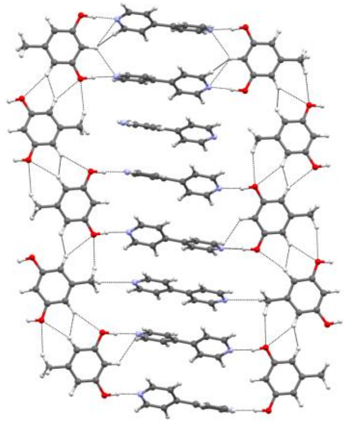

(d)

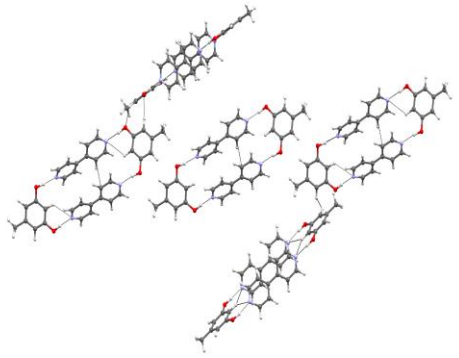

(e)

Fig. 2: Packing diagrams for (a) Form **I**; (b) Form **II**; (c) Form **III**; (d) Form **IV**; (e) Form **V**

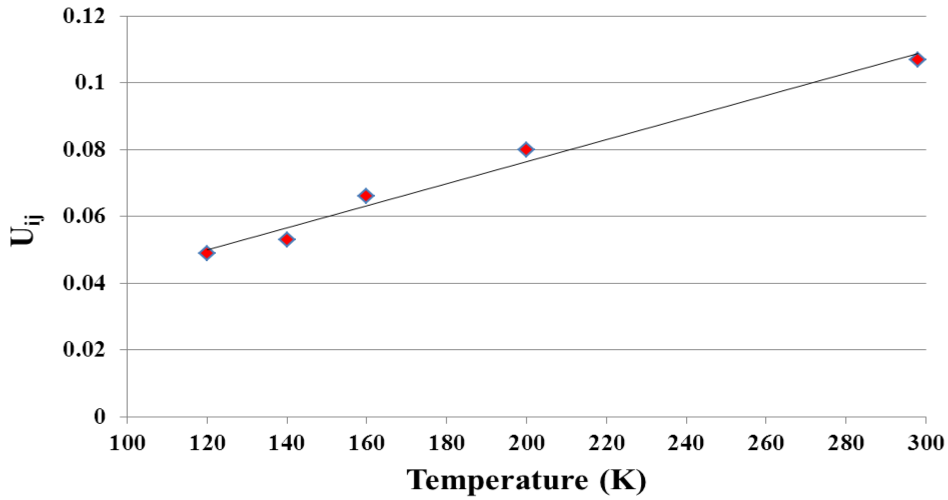

Fig. 3: Variation of U_{ij} with temperature in Form **III**

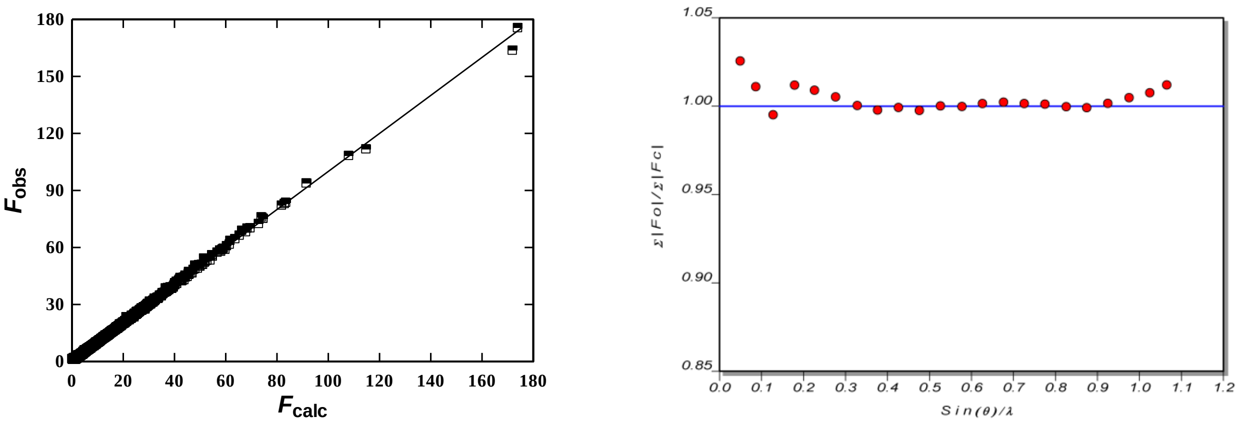

Fig. 4: Scatter plot depicting the variation of F_{obs} with F_{cal} for **4HYINA** (left) Variation of F_{obs}/F_{cal} with $(\sin\theta)/\lambda$ (right)

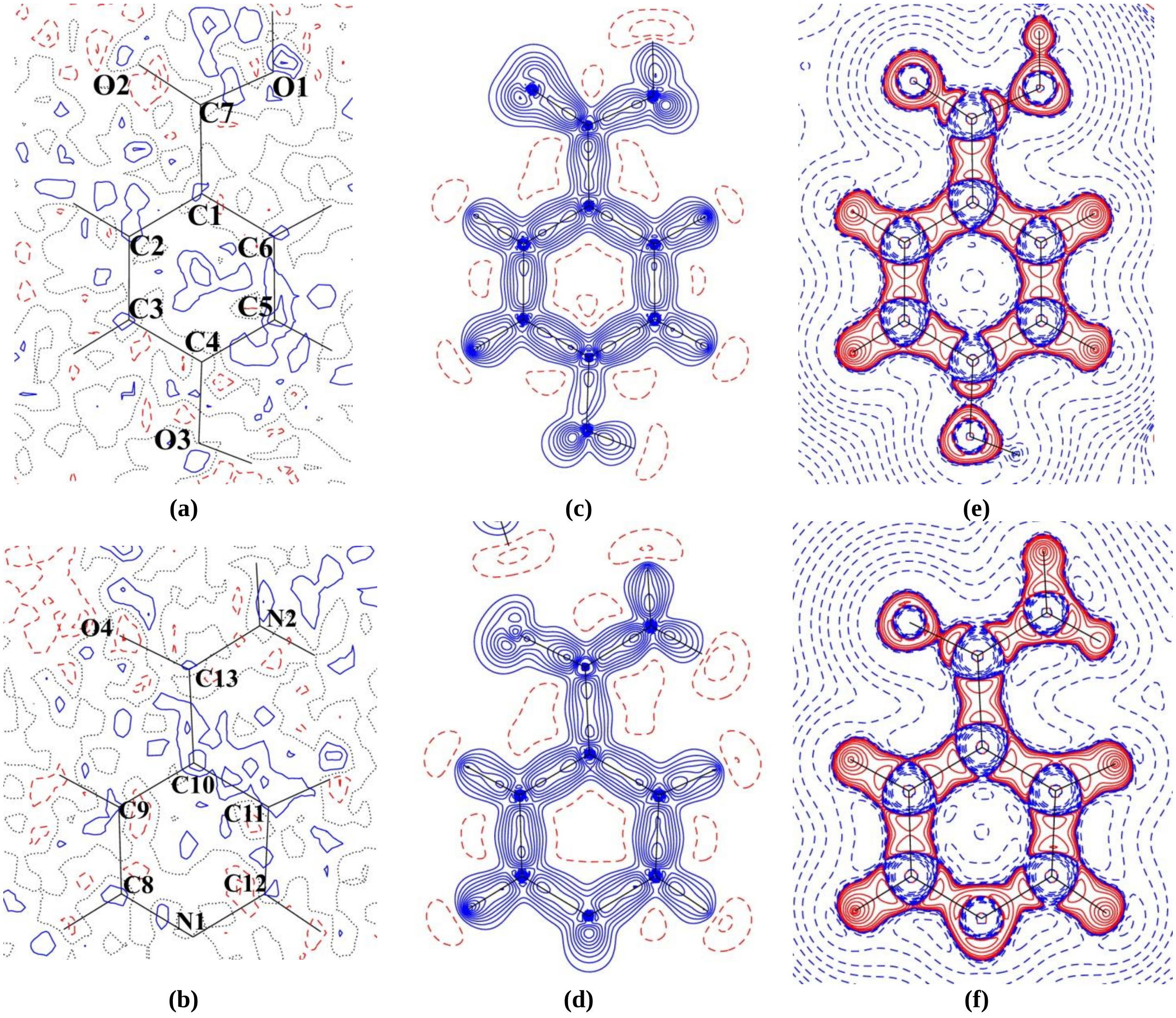

Fig. 5: (a, b) Experimental residual density (c, d) deformation density (e, f) Laplacian maps of 4-hydroxybenzoic acid: Isonicotinamide. Residual density contours are drawn at the intervals of ±0.05 e Å^{-3}. Deformation density contours are drawn at the intervals of ±0.1 e Å^{-3}. Laplacian plotted on logarithmic contours.

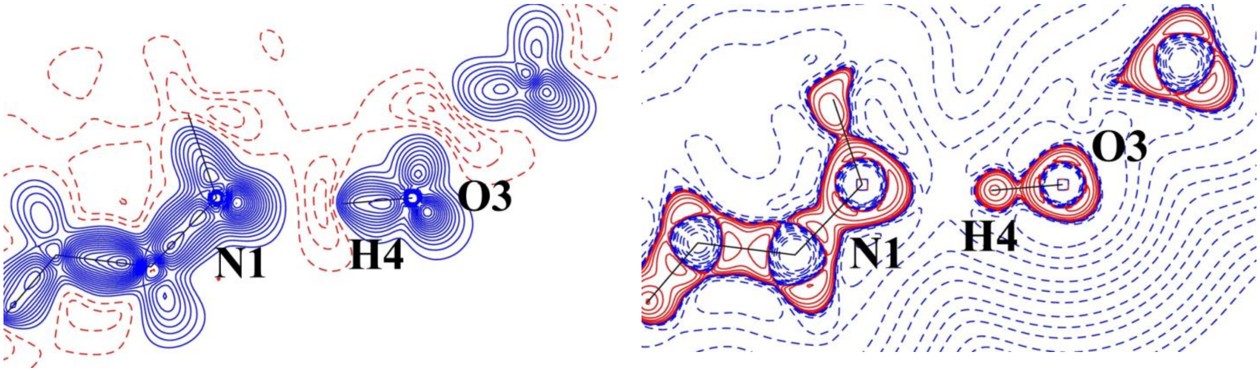

Fig. 6: Deformation density and Laplacian maps of intermolecular O–H···N synthon in 4-hydroxybenzoic acid: Isonicotinamide

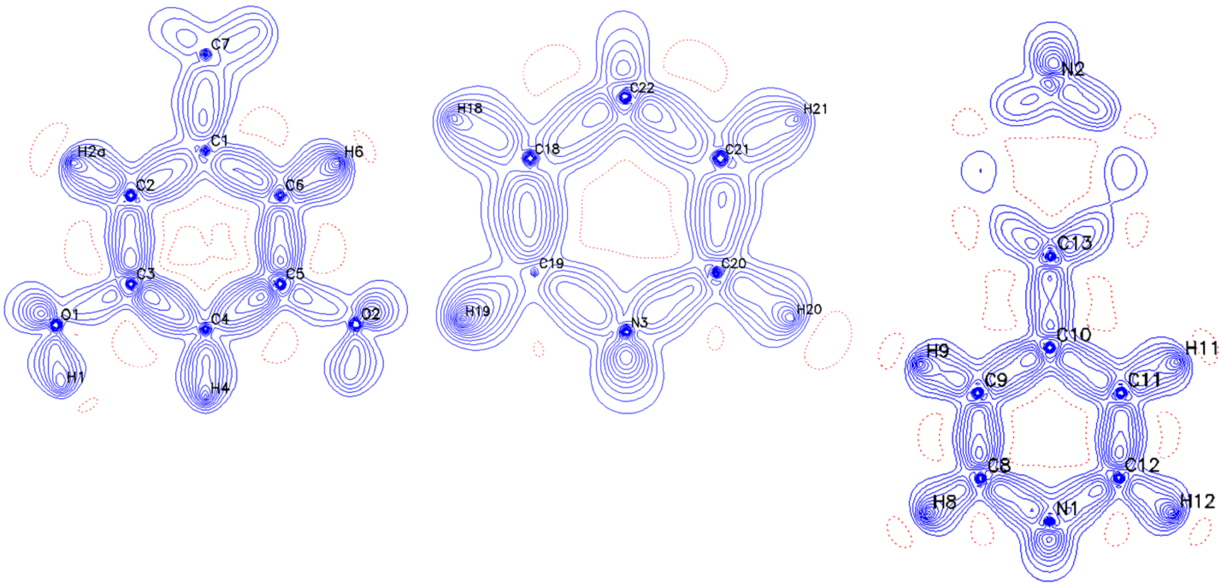

(a)

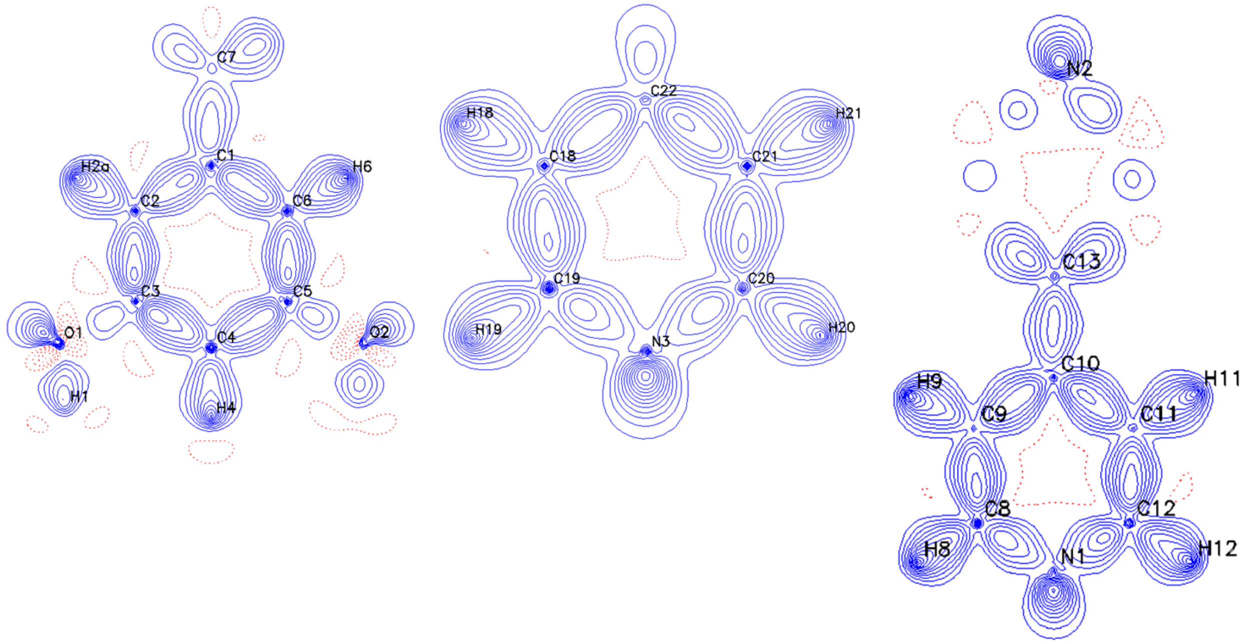

(b)

Fig. 7a: Comparison of 2D deformation density maps from (a) SBFA (b) Theory in Form **II**

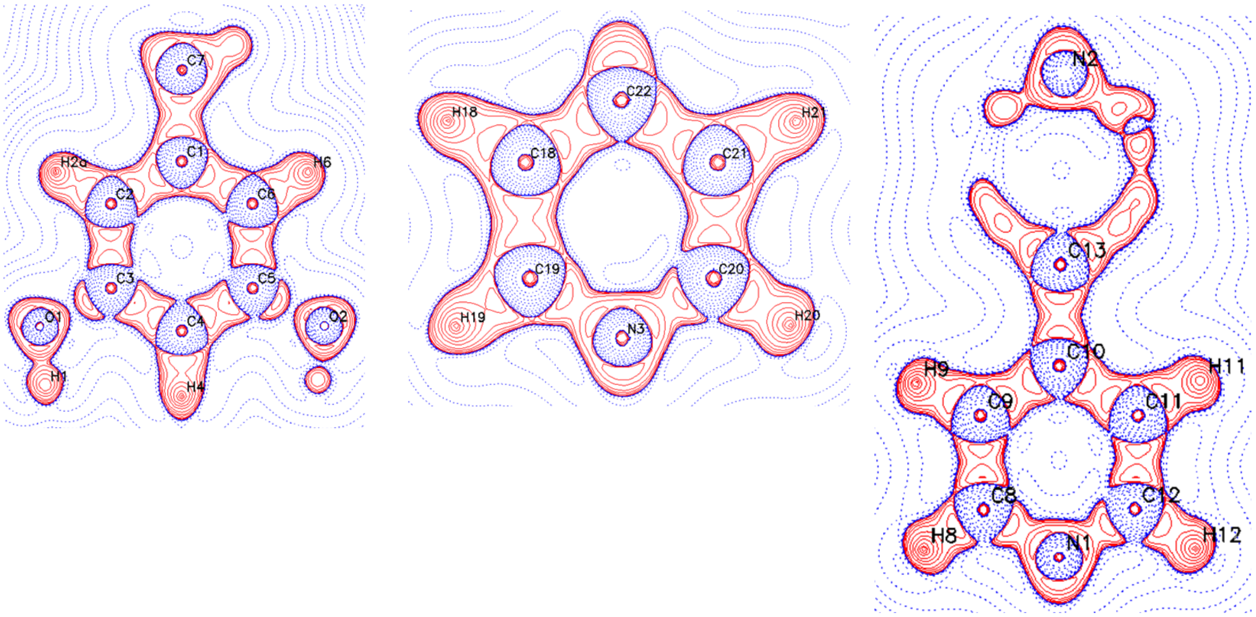

(a)

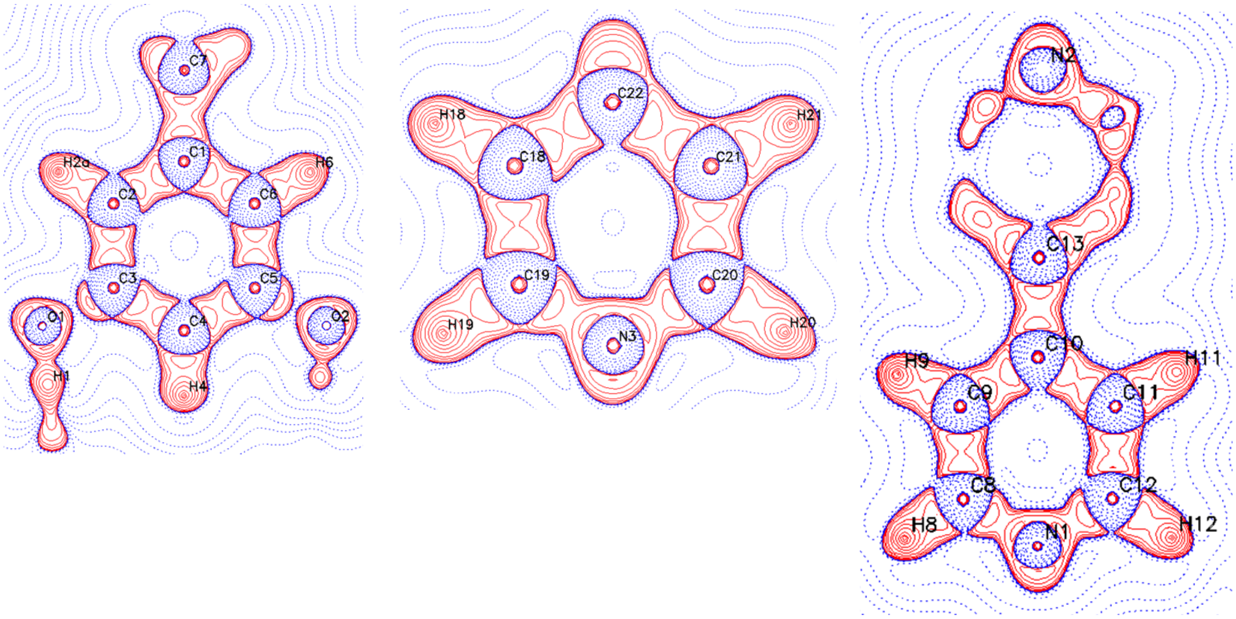

(b)

Fig. 7b: Comparison of 2D Laplacian maps from (a) SBFA (b) Theory in Form **II**

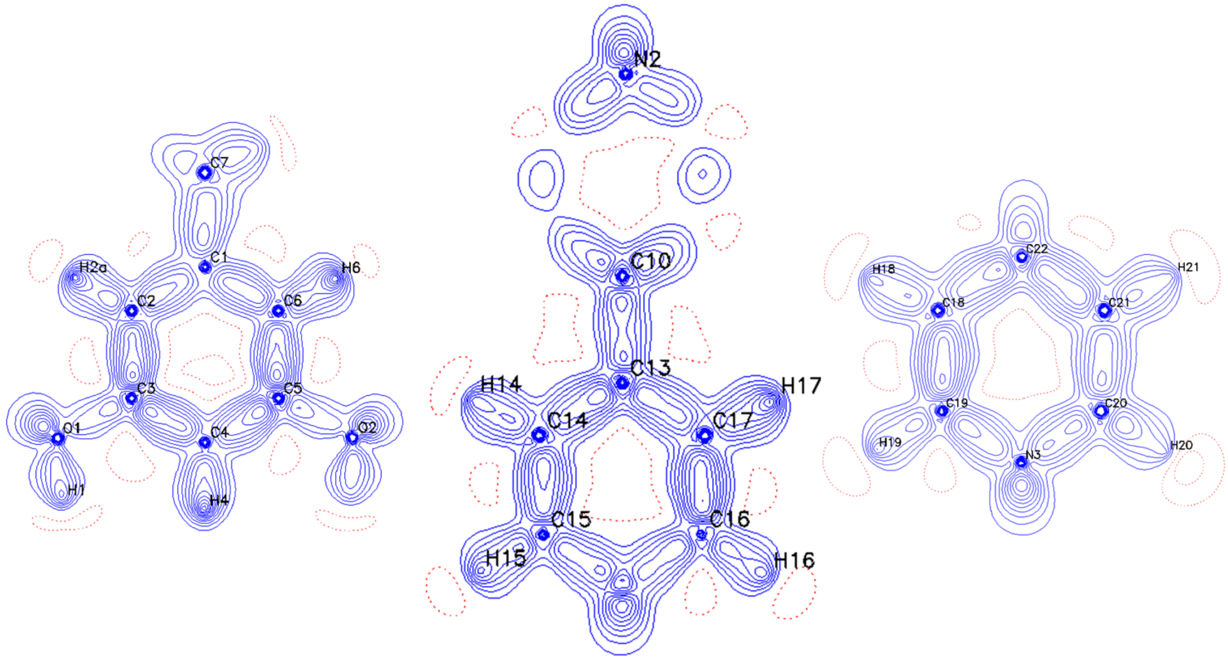

(a)

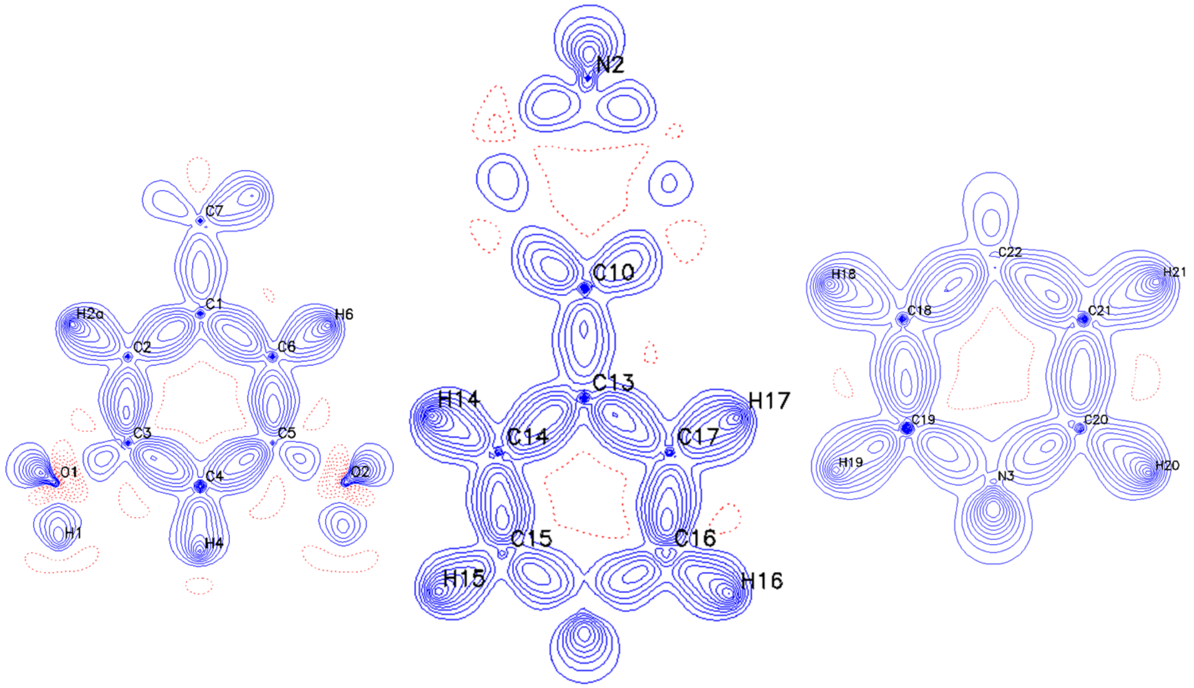

(b)

Fig. 8a: Comparison of 2D deformation density maps from (a) SBFA (b) Theory in Form **III**

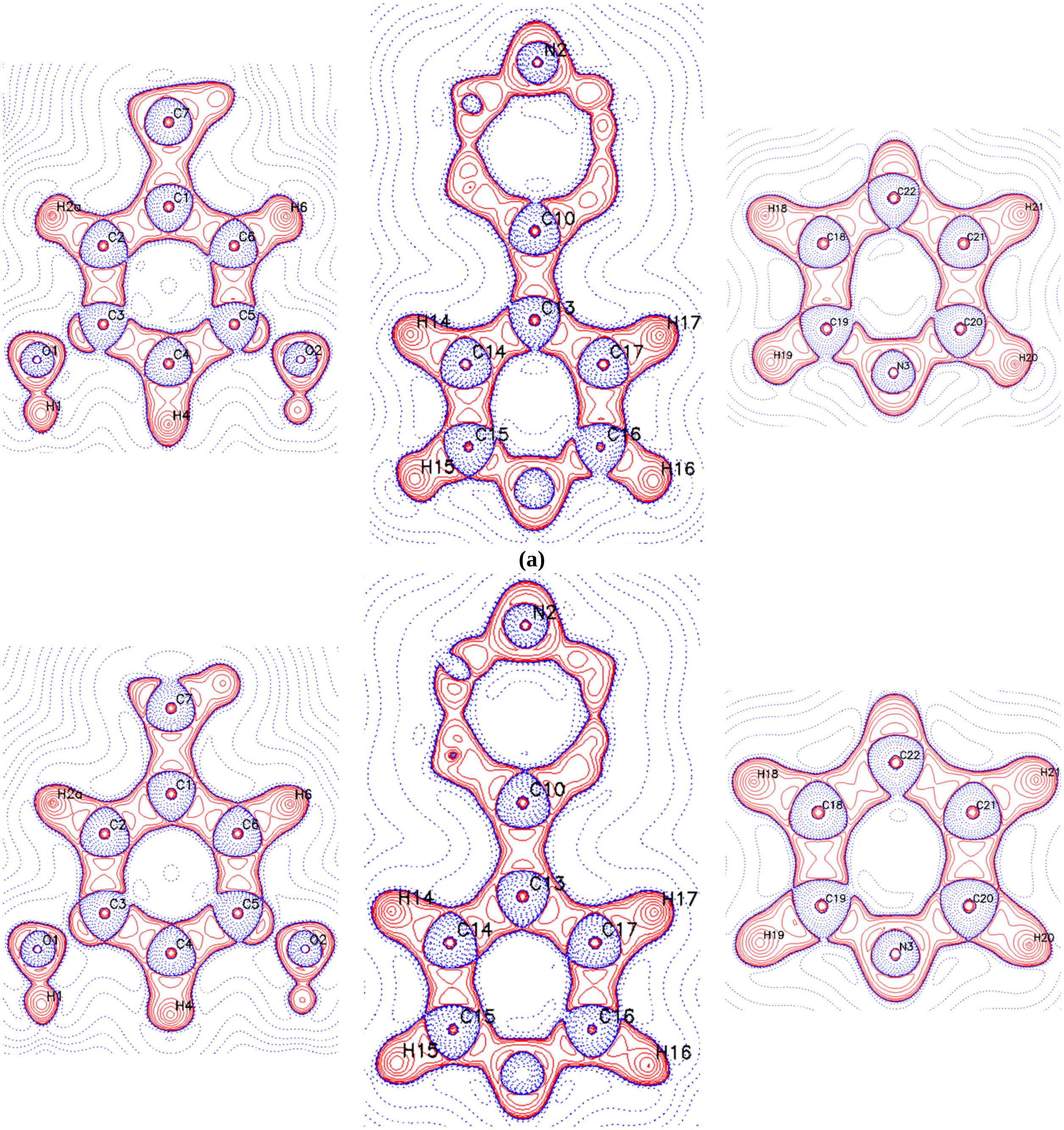

Fig. 8b: Comparison of 2D Laplacian maps from (a) SBFA (b) Theory in Form **III**

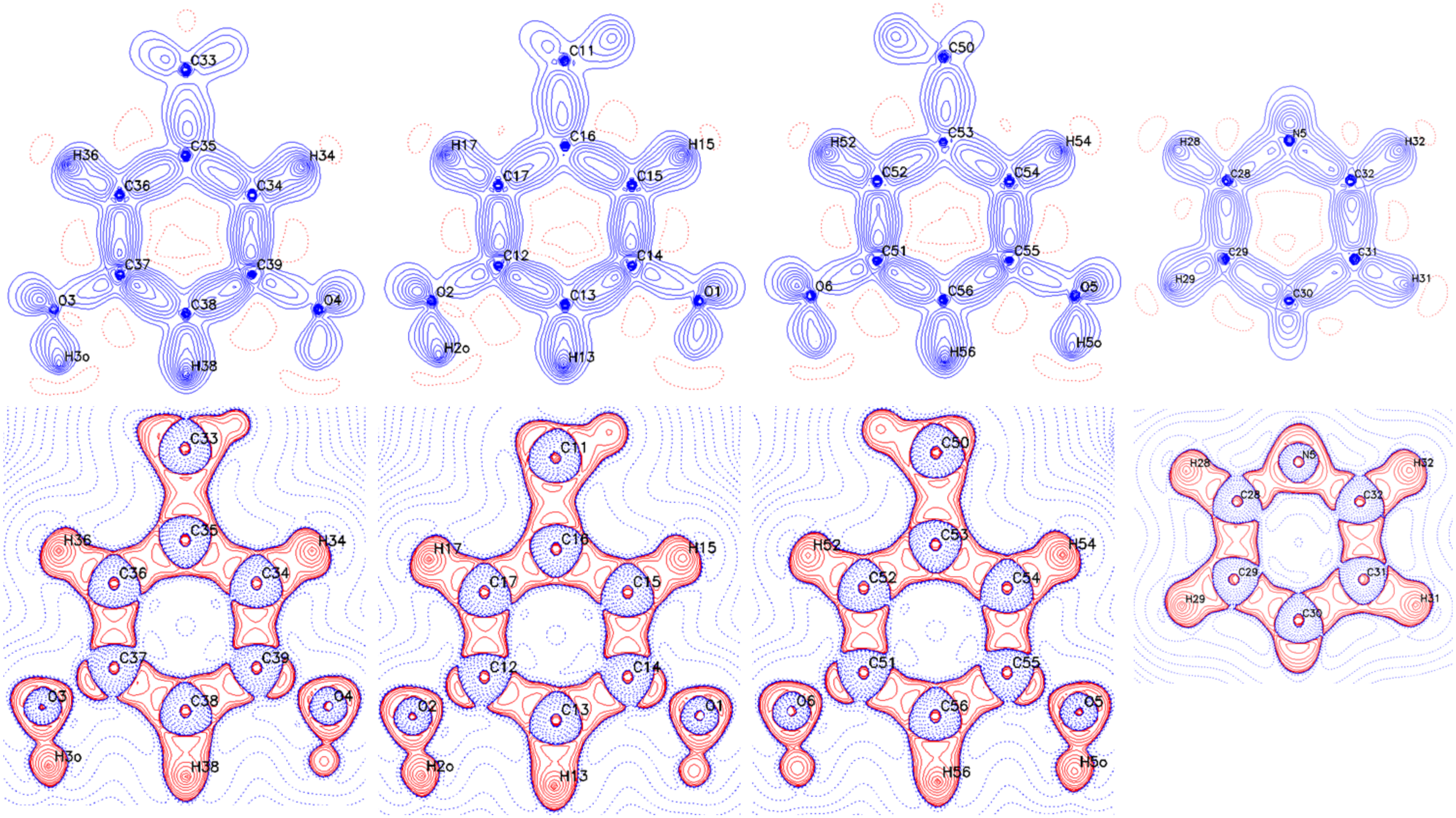

Fig. 9a: Deformation density and Laplacian maps of orcinols and half bipyridine in Form **IV** from SBFA.

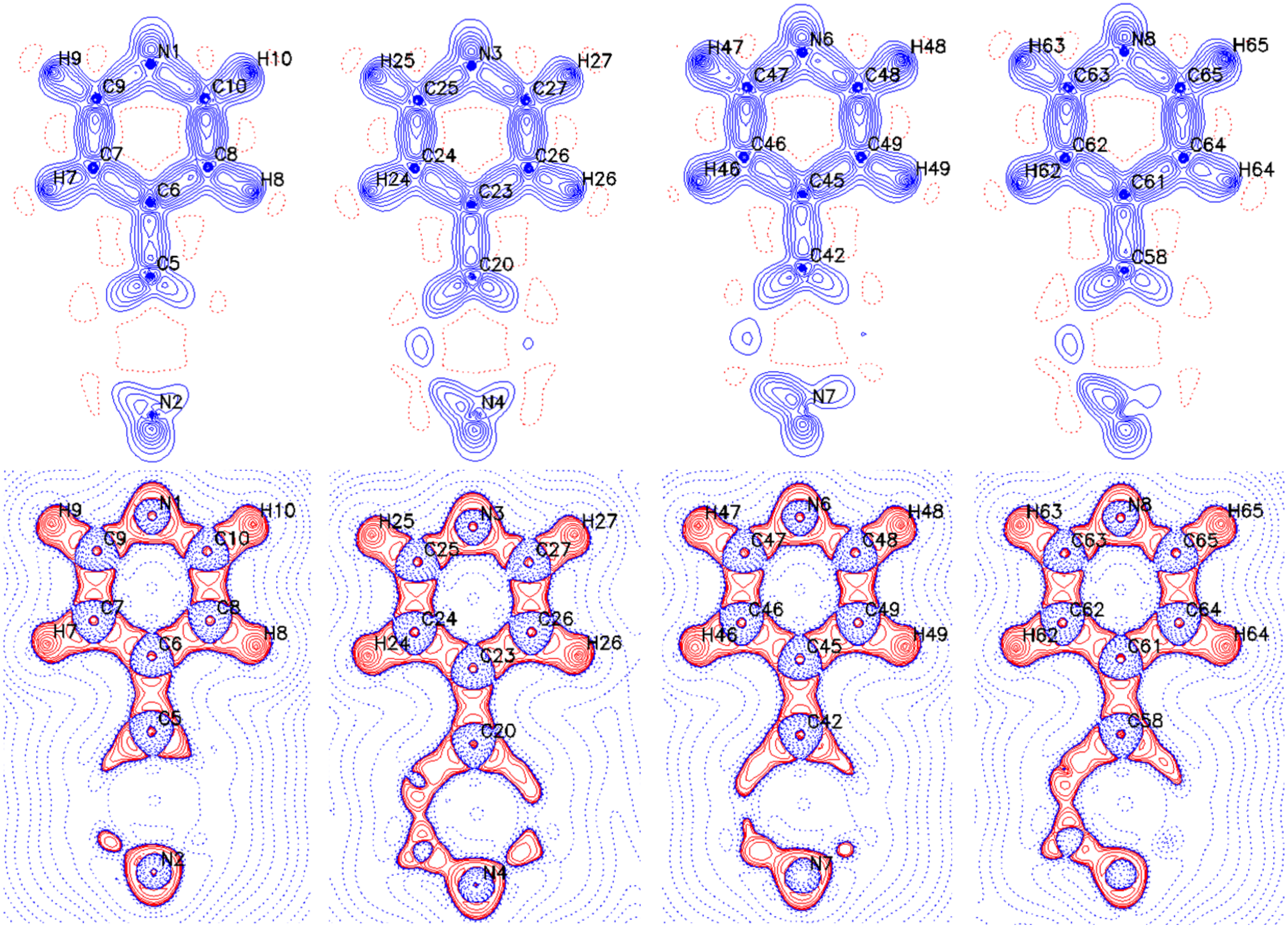

Fig. 9b: Deformation density and Laplacian maps of bipyridines in Form **IV** from SBFA.

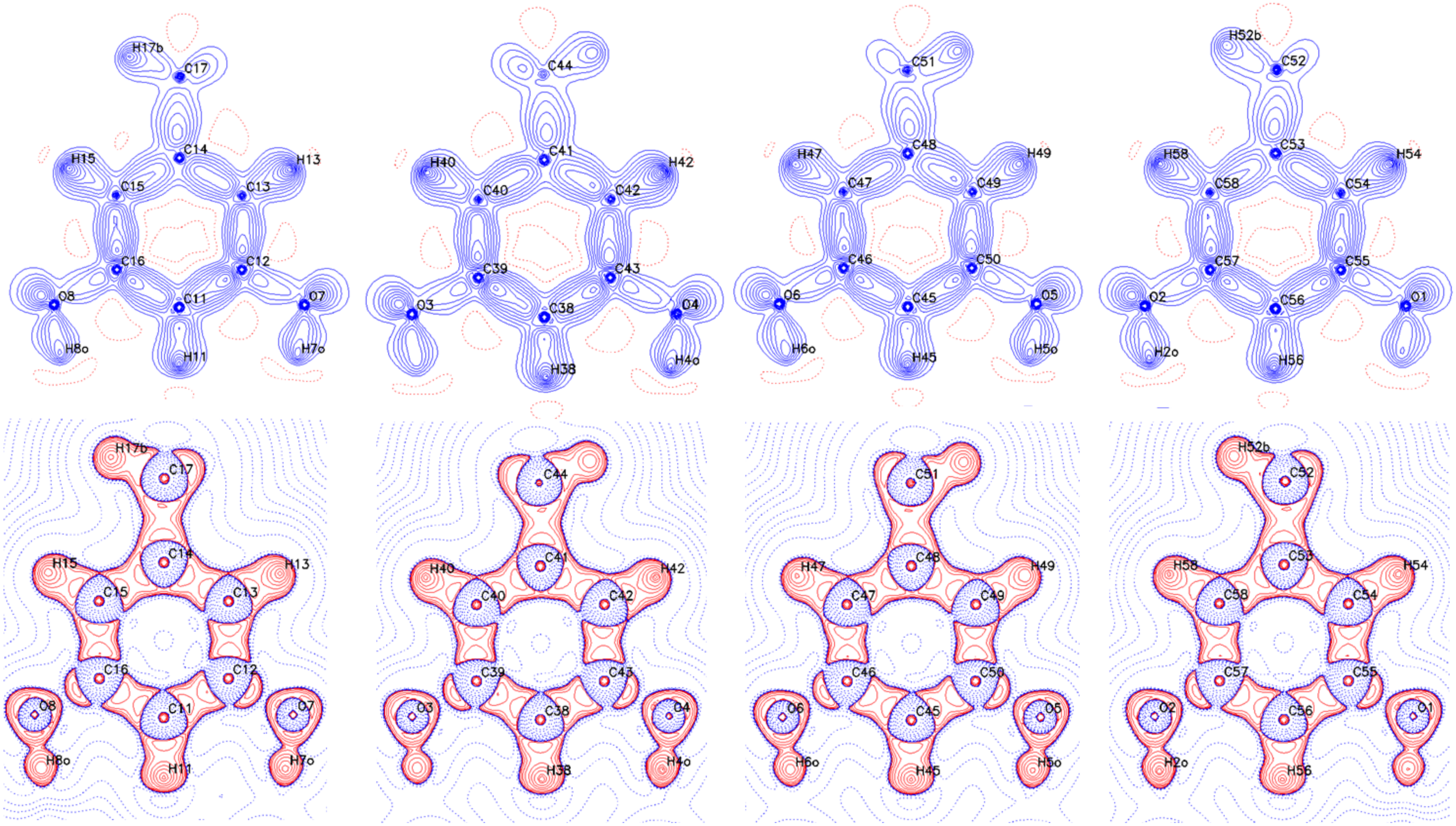

Fig. 10a: Deformation density and Laplacian maps of orcinols in Form **V** from SBFA.

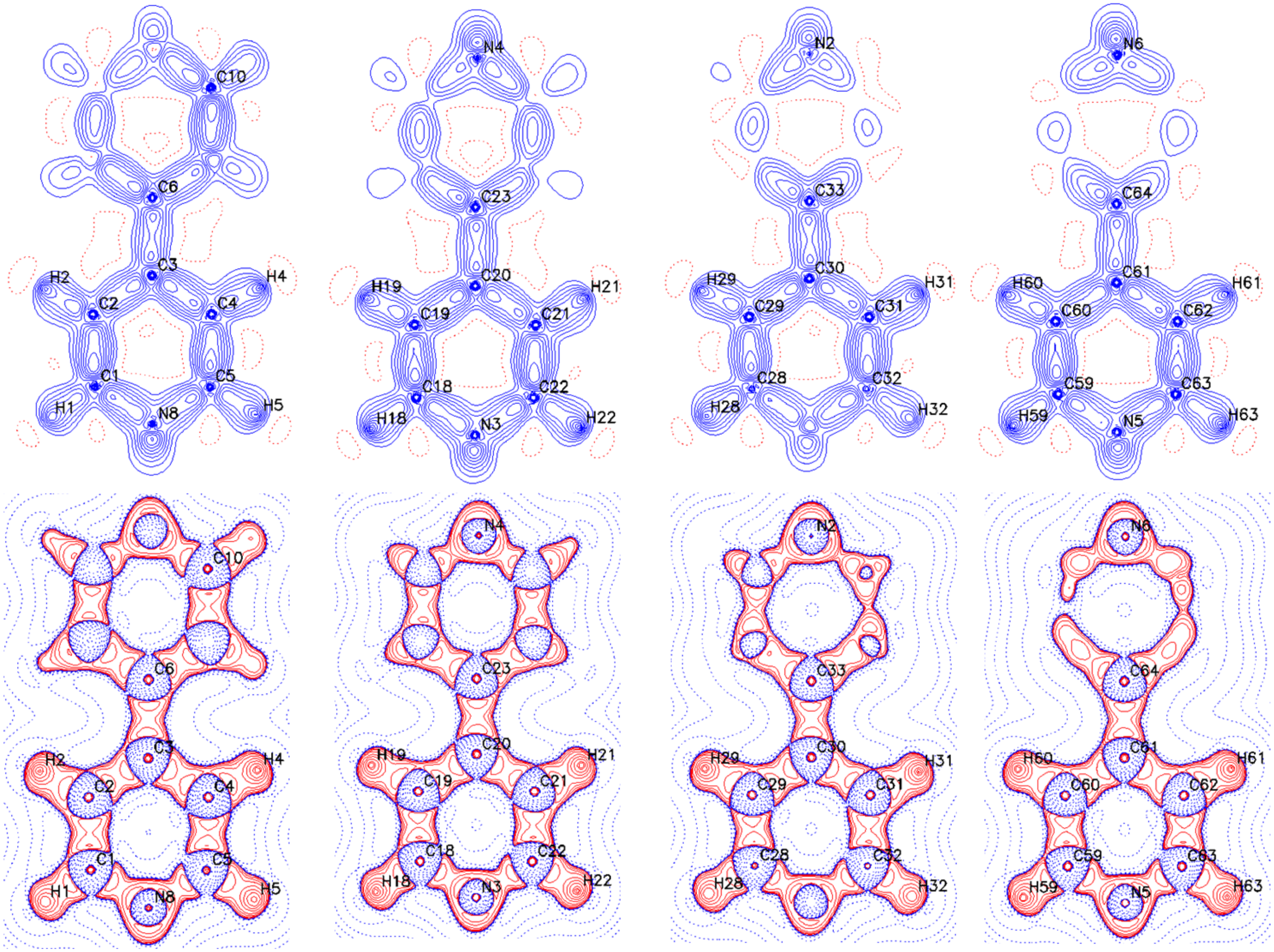

Fig. 10b: Deformation density and Laplacian maps of bipyridines in Form **V** from SBFA.

References:

Hathwar, V. R. & Guru Row, T. N. (2010). *J. Phys. Chem. A* **114**, 13434–13441.

Hathwar, V. R., Pal, R. & Guru Row, T. N. (2010). *Cryst. Growth Des.* **10**, 3306.

Hathwar, V. R. & Guru Row, T. N. (2011). *Cryst. Growth Des.* **11**, 1338.

Hathwar, V. R., Thakur, T. S., Guru Row, T. N. & Desiraju, G. R. (2011). *Cryst. Growth Des.* **11**, 616.

Hathwar, V. R., Thakur, T. S., Dubey, R., Pavan, M. S., Guru Row, T. N. & Desiraju, G. R. (2011). *J. Phys. Chem. A* **115**, 12852–12863.

Macchi, P. & Coppens, P. (2001). *Acta Crystallogr., Sect. A: Found. Crystallogr.* **57**, 656-662.

Madsen, A. (2006). *J. Appl. Crystallogr.* **39**, 757–758.

Munshi, P., Madsen, A. O., Spackman, M. A., Larsen, S. & Destro, R. (2008). *Acta Crystallogr., Sect. A: Found. Crystallogr.* **64**, 465–475.

Su, Z.; Coppens, P. (1998) *Acta Crystallogr., Sect. A: Found. Crystallogr.*, **54**, 357.

Volkov, A., Macchi, P., Farrugia, L. J., Gatti, C., Mallinson, P. R., Richter, T. & Koritsanszky, T. S. (2006). *XD2006.* Version 5.34. University at Buffalo, State University of New York, NY, USA,New York.
